# Supplementary material for: Top-down patterning of topological surface and edge states using a focused ion beam
Source: Nat Commun. 2023 Mar 27;14:1693. doi: 10.1038/s41467-023-37102-x (PMC10042877; doi:10.1038/s41467-023-37102-x)
Supplement: Supplementary file 1 — Supplementary Information [file 41467_2023_37102_MOESM1_ESM.docx]

**Supplementary Information**

**Top-Down Patterning of Topological Surface and Edge States using a Focused Ion Beam**

*Abdulhakim Bake^1,2*^, Qi Zhang^2,3*^, Cong Son Ho^4^, Grace L. Causer^5^, Weiyao Zhao^1,2^, Zengji Yue^6,7^, Alexander Nguyen^2,8^, Golrokh Akhgar^2,8^, Julie Karel^2,8^, David Mitchell^9^, Zeljko Pastuovic^10^, Roger Lewis^6^, Jared H. Cole^2,4^, Mitchell Nancarrow^9^, Nagarajan Valanoor^2,3^, Xiaolin Wang^1,2^, David Cortie^1,2,10 ϯ^*

*^1^ Institute for Superconducting and Electronic Materials (ISEM), University of Wollongong, Wollongong, NSW 2522, Australia*

*^2^ The Australian Research Council Centre for Excellence in Future Low Energy Electronics Technologies*

*^3^ School of Materials Science and Engineering, The University of New South Wales, Kensington, New South Wales 2052, Australia*

*^4^ Chemical and Quantum Physics, School of Science, RMIT University, Melbourne, Australia*

*^5^ Physics Department, Technical University of Munich, 185748 Garching, Germany*

*^6^ School of Physics,* *Faculty of Engineering and Information Science, University of Wollongong, Wollongong, NSW 2522, Australia*

*^7^ Institute of Photonic Chips, University of Shanghai for Science and Technology, Shanghai, 200093, China*

*^8^ Department of Materials Science and Engineering, Monash University, Clayton, VIC 3800, Australia*

*^9^ Electron Microscopy Centre, University of Wollongong, Wollongong, NSW 2522, Australia*

*^10^ The Australian Nuclear Science and Technology Organisation (ANSTO), Lucas Heights, NSW 2234, Australia*

*^ϯ^ Corresponding author email:* [*dcortie@uow.edu.au*](mailto:dcortie@uow.edu.au)

**Both of the first authors contributed equally to this work*

# The penetration depth of the gallium ions into Sb_2_Te_3_

## Monte Carlo calculations

The results in the main manuscript show experimentally how irradiation with Ga ions modifies the atomic structure and electronic properties of a Sb_2_Te_3_ surface. This section of the Supplementary presents complementary Monte Carlo (MC) calculations to clarify the nature of the ion-beam/matter interaction, and radiation damage, for Ga implanted into Sb_2_Te_3_.

The Stopping and Range of Ions in Matter (SRIM)^1^ software was used to simulate the characteristics of Ga^+^ ions implanted into a Sb_2_Te_3_ surface. Supplementary Figure 1 (a) shows that the collisions taking place within the energy range of 8-40 keV (as in the experiment) are dominated by nuclear energy loss (i.e., via phonons and ionic displacements rather than via electronic excitations). This is a characteristic of low-energy ions and is quite distinct from the energy loss of high-energy ions in the MeV range, where electronic loss is the primary mechanism. Ultimately, the low-energy Ga^+^ ions penetrate only several nanometers before losing sufficient kinetic energy to become stationary. Supplementary Figure 1 (b) shows the projected ion range as a function of the initial kinetic energy. Incident ions with 8–40 keV have a mean penetration depth of 7–23 nm. Supplementary Figure 1 (c) shows that the longitudinal and lateral straggle is comparable in the 5–20 nm range. For an ideal parallel-collimated ion beam and infinitely sharp mask, the lateral implant profile would be slightly tighter than the vertical spread. However, in a focused ion beam microscope, the beam spot is typically ~100 nm wide with a 3D Gaussian profile (see Supplementary Section 1.3). Thus, in practice, for the experiments performed in this work, the actual interaction volume of the ion beam is larger in the lateral dimension than in the vertical dimension.


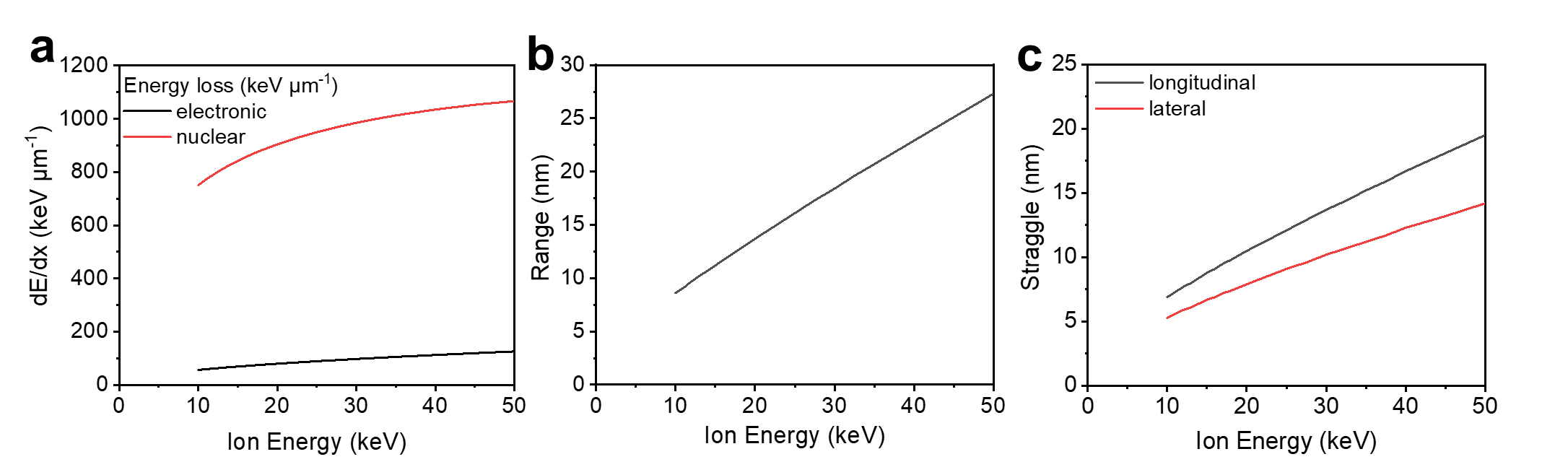


Supplementary Figure 1. Ga^+^ ions stopping range and energy loss in Sb_2_Te_3_. (a) The energy loss for various incident ion energies. (b) The projected ion range as a function of the incident ion energy. (c) The longitudinal and lateral straggle.

To evaluate the effect of ion-beam damage introduced by the incident Ga^+^ ions, the damage-per-atom (DPA) profiles were calculated for the Sb_2_Te_3_ using the Supplementary Equation (1), as in past work^2^:

$DPA=\frac{N_{d}FM}{\rho N_{A}}$ Supplementary Equation (1)

where *N_d_* is the number of Sb/Te displacements per ion calculated by the SRIM software (where this quantity varies as a function of depth), *F* is the Ga ion fluence, *M* is the molar mass, ρ is the mass density of the target material (Sb_2_Te_3_) and *N_A_* is the Avogadro number. In general, as shown in Supplementary Figure 2 (a)-(d), the total damage in the Sb_2_Te_3_ crystal increases with the Ga ion fluence, with Sb and Te atoms being displaced between 1–60 times per incident Ga ion for fluences between 1x10^15^ and 1x10^16^ ions/cm^2^. Apart from the ion species and fluence, the angle of incidence and energy are other important factors governing the damage depth and sputter rate. In the present work, the Sb_2_Te_3_ crystals were always irradiated with the beam perpendicular to the surface. The total damage increases with the ion beam energy for the same ion fluence in Supplementary Figure 2 (a)-(d), given the additional nuclear energy loss required. The sputtering rate is determined by the nuclear stopping collisions and by the surface binding energy of the Sb and Te atoms which are removed by the sputtering process. Due to the low sputtering yield for Ga:Sb_2_Te_3_, sputtering only becomes significant at higher fluences and implantation is the dominant process at lower fluences^3^. For the regime where sputtering is negligible, Supplementary Figure 2 (e)-(h) shows the predicted Ga concentration profile (in atomic percent) as a function of the depth (from the top surface of the crystal) superimposed on the DPA profile calculated for the Ga ion dose at 10^15^/cm^2^ for energies in the range of 8–40 keV. According to the Ga concentrations calculated from SRIM (Supplementary Figure 2) and the experimental EDS analysis in the later section (Supplementary Figure 3), the typical amount of Ga in the amorphous regions of the sample is 0.5–2 atomic percent for the fluences used in this work.


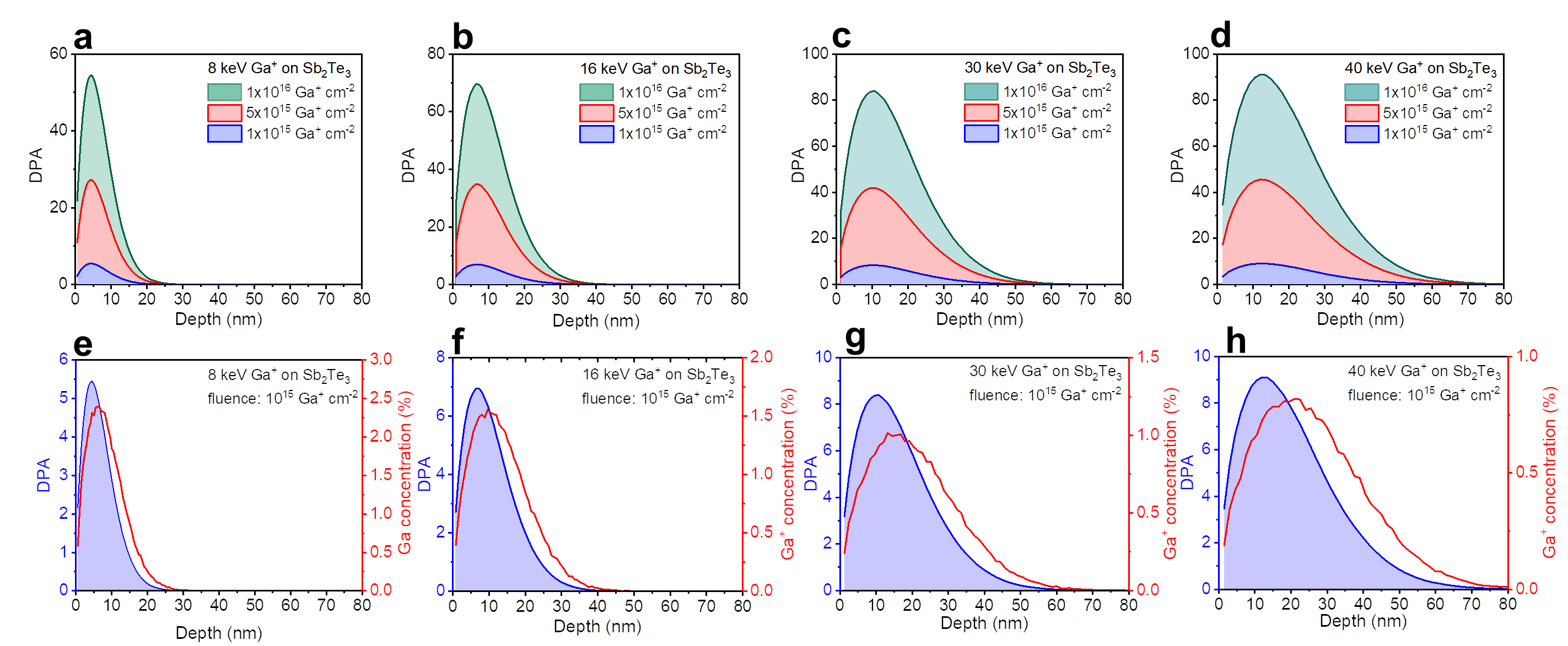


Supplementary Figure 2. Monte Carlo calculations of the ion-beam irradiation process in Sb_2_Te_3_ showing the Ga concentration and total damage profiles. (a)-(d) Accumulated damage profiles for Ga ion beam energies between 8–40 keV for the selected fluences of 10^15^, 5×10^15^, and 10^16^ ions/cm^2^, respectively. (e)-(h) The variation of the Ga concentration in Sb_2_Te_3_ at Ga ion beam energies between 8–40 keV for the selected fluence of 10^15^/cm^2^_._

## Validation of the ion beam stopping depth calculations against STEM images

In this section, scanning transmission electron microscopy (STEM) was used to experimentally study a Sb_2_Te_3_ crystal surface exposed to an 8 keV Ga ion beam with 2.18×10^15^ ions/cm^2^ (3.5×10^-12^ C/µm^2^) fluence. The procedure for high-quality FIB lamella preparation from Sb_2_Te_3_ single crystals can be found in our publication^4^. The STEM characterizations were performed on cross-sections of the surface to study the depth-dependency of the damage profile. These datasets were combined with complementary electron back-scattering diffraction measurements in a scanning electron microscope (SEM) to determine the lateral feature dimensions. These measurements enable a comparison with the damage depths obtained from Monte Carlo calculations described in Supplementary Section 1.1.

Supplementary Figure 3 (a)-(c) shows that the 8 keV beam penetrated to an approximate depth of 20–30 nm below the surface and caused amorphization. The maximum penetration depth is in good agreement with the earlier Monte Carlo theory calculations (Supplementary Figure 2 a, e). Several images at various magnifications are presented to survey the region where the Ga beam impacted the surface. At high magnifications, as in Supplementary Figure 3 (a), clear lattice fringes from the quintuple layers of Sb_2_Te_3_ can be observed in the STEM images in the unirradiated regions, confirming that the surface was initially crystalline. The images are taken in high-angle annular darkfield (HAADF) mode so that the intensity originates from atomic contrast, and secondary diffraction effects are minimal. A lower magnification image of the region impacted by the beam is shown in Supplementary Figure 3 (b). In both images, the amorphous region appears as a featureless dark grey area. A remarkably sharp interface is formed between the amorphous and single-crystalline Sb_2_Te_3_ below the central irradiated region (Supplementary Figure 3 (a)), directly below the point where the amorphization depth is greatest. In contrast, the STEM images in Supplementary Figure 3 (g) and (h) are taken at the lateral edges of the irradiated region (as indicated in Supplementary Figure 3 (c)). These show that the amorphous/crystalline interface on the lateral borders of the beam spot is not as sharp. This is attributed to the Gaussian spread of the focused ion beam spot (laterally), which causes an intensity gradient on the edges of the beam spot.

To enable a side-by-side comparison of theory and experiment in the vertical dimension, Supplementary Figure 3 (f) shows the DPA and Ga atomic percentage profiles in Sb_2_Te_3_ for the Ga^+^ ions with 8 keV energy and the fluence of 10^15^ ions/cm^2^ calculated from SRIM. This can be compared with the experimental EDS profile (Supplementary Figure 3 (e)). The Ga atomic percentage variation in Supplementary Figure 3 (e) is very low in the irradiated regions, below 2±1.5 atomic %, in agreement with the MC predictions. Indeed, the Ga concentration is low enough to be near the detection limit with the EDS technique, and there is considerable uncertainty in the experimental measurement. Although the final concentration of Ga is low, the main effect of Ga irradiation is to cause displacements of Sb/Te atoms (up to 60 displacements per Ga atom). Thus, the STEM images clearly show the after-effects of the irradiation (Supplementary Figure 3 g/h), where the amorphous phase is formed by the beam damage, precisely in the region accessed by the Ga ions during the irradiation. In addition, a faint elemental signal is also visible in the two-dimensional EDS elemental mapping in Supplementary Figure 3 (d) (which probes the same region of interest shown in Supplementary Figure 3 (c)). The depth of the damaged region and the region enriched with Ga agree well with the profile predicted by the Monte Carlo calculations (Supplementary Figure 3 (f)).


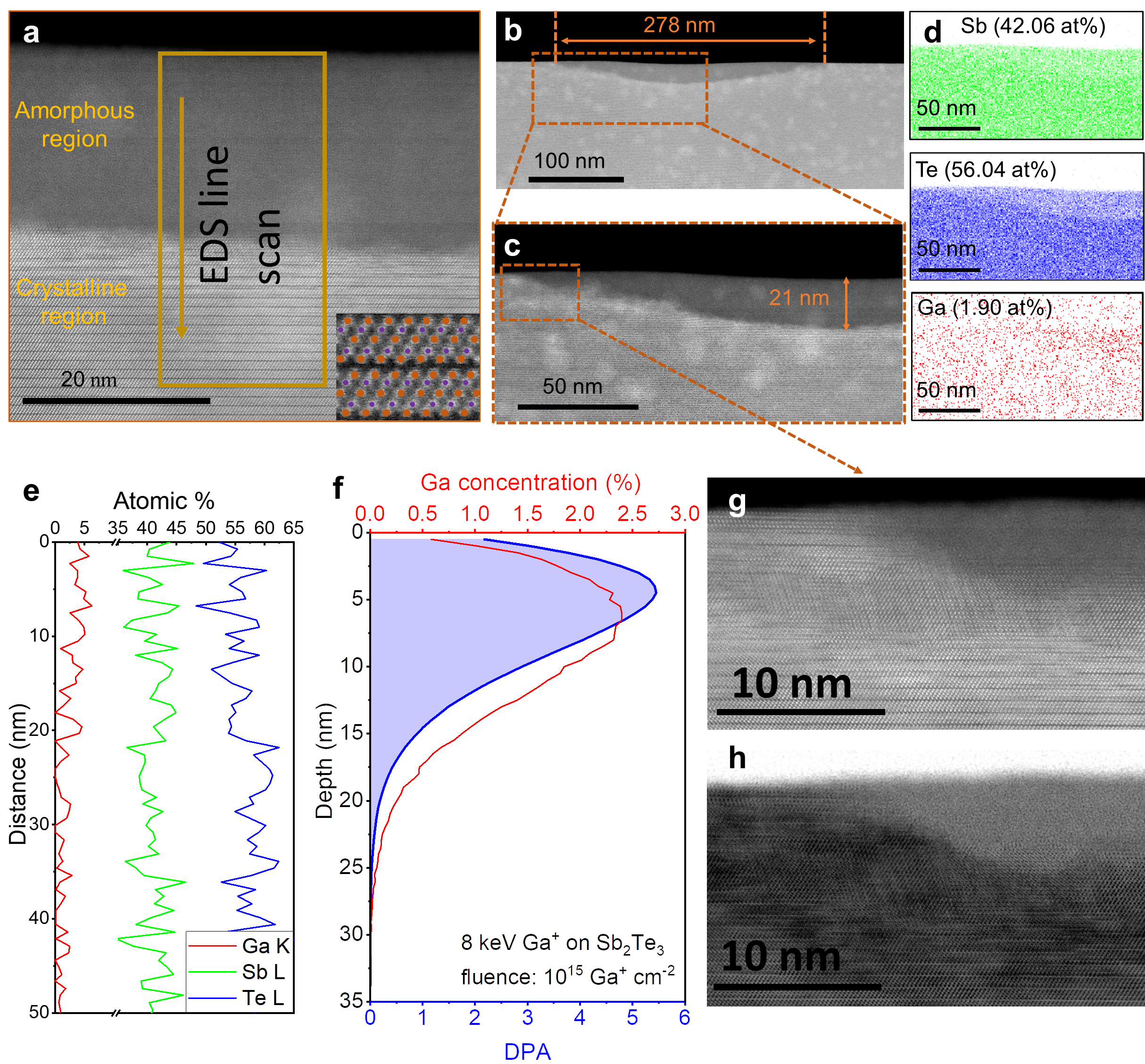


Supplementary Figure 3. (a) STEM HAADF image showing distinct amorphous and crystalline regions. The inset shows an enlarged image of the quintuple atomic layered structure of Sb_2_Te_3_ superimposed with Sb and Te atoms. (b) Low magnification image surveying the cross-section of the Ga-irradiated region of the Sb_2_Te_3_ surface. The ion beam energy was 8 keV, and the fluence was 2.18×10^15^ ions/cm^2­^. (c) Detail of the boxed region in b) showing the lateral and depth scale of the amorphous region near the edge of the irradiated area. (d) EDS elemental maps of (c). (e) Ga, Sb, and Te line profiles are taken along the line shown in (a). (f) Monte Carlo calculations of the displacement per atom and Ga atomic concentration within Sb_2_Te_3_ for 8 keV Ga^+^ ions at a fluence of 10^15^ ions/cm^2^. (g) HAADF image showing a detail of the boxed region in (c). (h) Bright-field image of (g) showing strong diffraction contrast (dark) from the crystalline regions, which were not exposed to the Ga ion beam.

## Lateral feature size from the ion beam writing process

The damaged region takes the form of a 3D Gaussian as determined by the lateral beam shape and horizontal straggle, together with the vertical penetration depth. To illustrate this point, Supplementary Figure 4 (a) shows the 3D distribution profile of Ga^+^ ions and the intrinsic horizontal straggle predicted by the Monte Carlo calculations for an infinitely sharp beam. In the experimental case, the horizontal dimension deviates from the theoretical calculations because the ion beam is Gaussian with a width of ~ 100 nm. To assess the lateral sharpness of the pattern in the horizontal dimension, Supplementary Figure 4 (b) shows a secondary electron image of the Sb_2_Te­_3_ single crystal surface after irradiation with a Ga focused ion beam to form a series of parallel lines. The nominal widths of these irradiated regions specified in the input pattern were 20, 50, and 100 nm. The corresponding EBSD band contrast image of Supplementary Figure 4 (c) shows the ion beam-induced amorphization as indicated by dark lines on the image. From further analysis of the gray values on the band contrast image, we have calculated the approximate width of the Ga ion beam. Gaussian peak fitting was used to obtain the FWHM of each peak of 166, 184, and 220 nm, respectively, as shown in Supplementary Figure 4 (d).


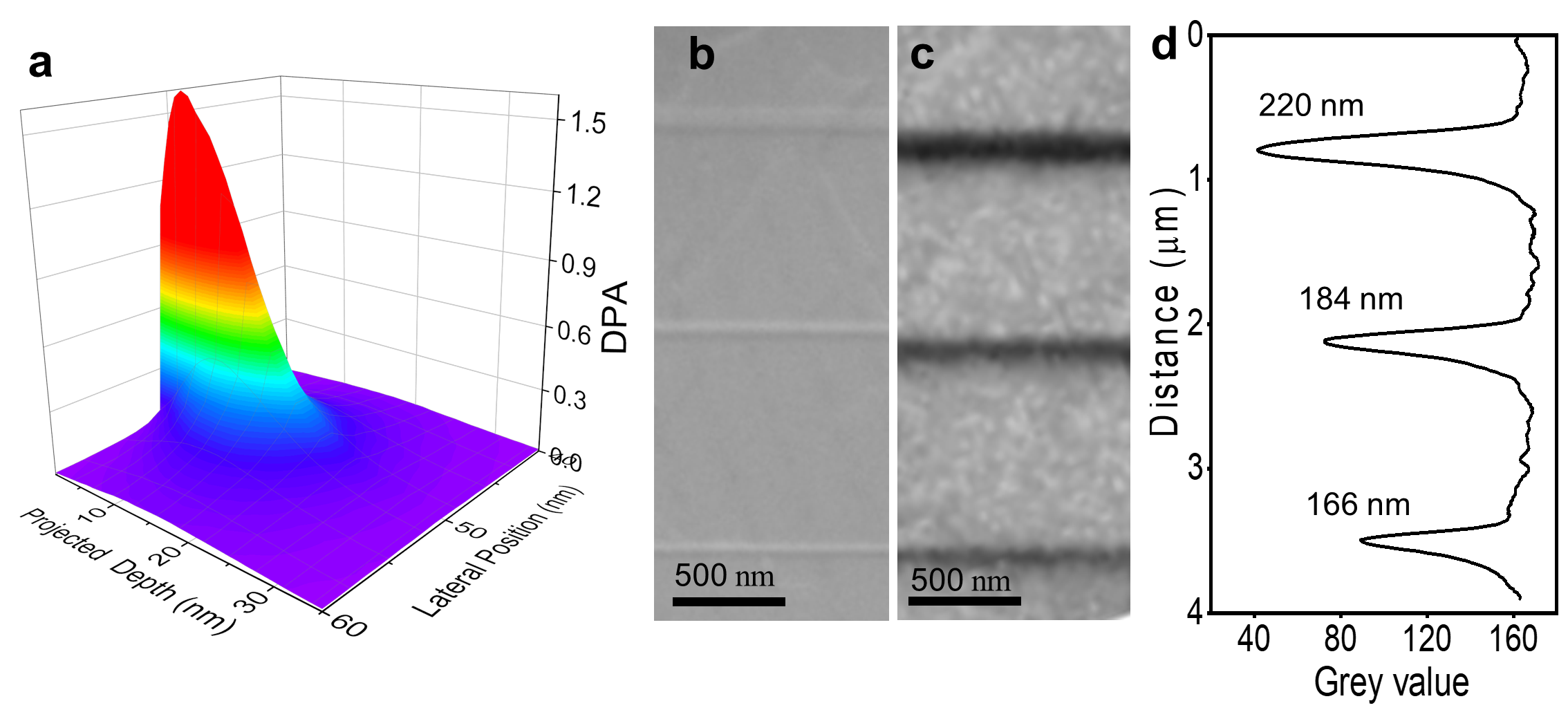


Supplementary Figure 4. (a) 3D distribution of the Damage-Per-Atom profile in a Sb_2_Te_3_ crystal for a Ga beam with energy 8 keV, as calculated from the Monte Carlo calculations. (b) Secondary electron image showing Ga ion beam irradiated lines of various widths (top to bottom, input pattern width of 100, 50 and 20 nm) and (c) corresponding EBSD band contrast image. (d) Vertical intensity profile through the band contrast image measures the actual width of the amorphized regions as (top to bottom), 200, 184 and 166 nm.

## The crystallinity of the underlying Sb_2_Te_3_ crystal after low-energy ion implantation

To confirm the orientation and crystallinity of the Sb_2_Te_3_ pieces used for FIB fabrication, X-ray diffraction (XRD) was conducted with a Rigaku XRD SmartLab using a Cu K_α_ source. The crystal was mounted with the (00L) direction along the scattering vector in Bragg-Brentano geometry. A 2D detector was used to collect the pattern, and the central region of the detector was integrated to yield the 1D pattern shown in Supplementary Figure 5. The observed peaks obey the selection rule (00L) where L=3n and n is an integer, consistent with the R-3c space group for Sb_2_Te_3_, and the peaks have been indexed using the Inorganic Crystal Structure Database (ICSD Entry #192780). As the X-ray penetrates many micrometers into the sample, the XRD in this geometry is dominated by the crystalline region underneath the nanometer-scale implanted region at the surface. In Supplementary Section 4.5, complementary data is given using low-angle (reflectometry) measurements for thin films to probe the implanted region directly.


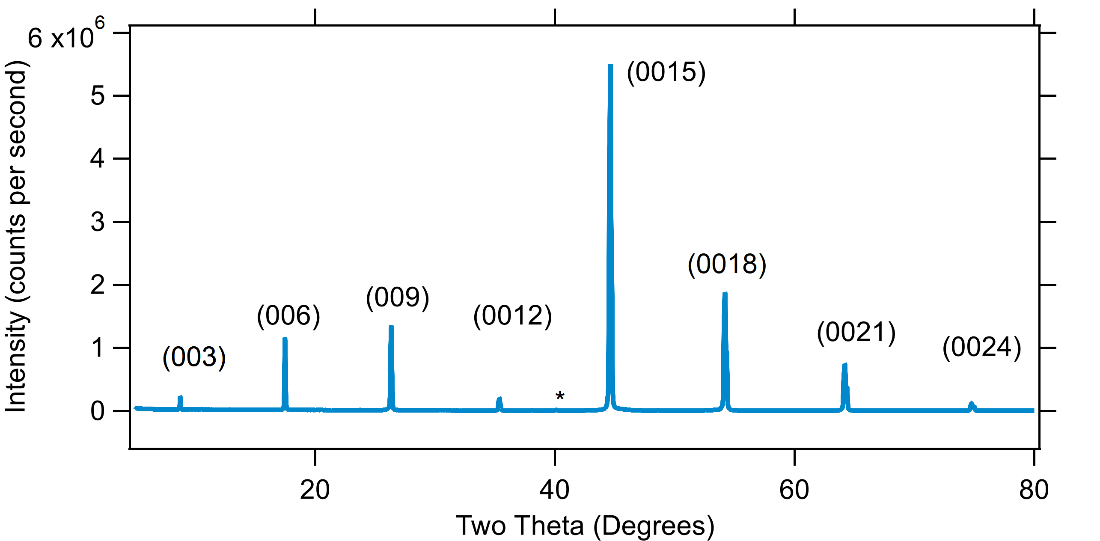


Supplementary Figure 5. The XRD diffraction pattern of a c-axis orientated Sb_2_Te_3_ crystal was irradiated with 1x10^17^ /cm^2^ Ga ions at 40 keV. The strong (00L) diffraction peaks show that the crystallinity is preserved below the nanometer-scale implanted region. The much smaller peak marked with a* is from Cu K_β_.

# Additional data for the effect of irradiation fluences and geometries

## High fluence irradiation leading to sputtering

This section presents additional electron back-scattering diffraction (EBSD) and AFM measurements of Sb_2_Te_3_ surfaces. The data is presented for a wider variety of ion-beam fluences compared to the datasets in the main text, which focused only on a subset. These show the additional effects of the ion beam on laterally patterned surface regions via amorphization and forming trenches/valleys (i.e. sputtering at higher fluences).

The patterns in Supplementary Figure 6 (a) were formed using a single ion beam energy of 8 keV and a constant current of 1.9 pA. The irradiation time was varied in each square to modify the overall fluence. The ion fluence of each region (R1-R9) is provided in Supplementary Table 1. The higher fluences in regions R7, R8, and R9 produce clear shallow trenches, which indicates the onset of material removal by the sputtering process (Supplementary Figure 6 (a)) and quantified using atomic force microscopy (Supplementary Figure 6 (b)). The trenches are very shallow in this fluence regime (<10 nm). For lower fluences, the Sb_2_Te_3_ crystals experience slight swelling due to Ga implantation giving a nanometer-scale increase in surface height. This transition from swelling to sputtering occurs around 10^15^ ions/cm^2^ for the 8 keV beam, and a similar transition was reported for Ga irradiation in silicon^3^.

EBSD scans were performed for the region of interest using an electron beam voltage of 20 keV and a current of 2.8 nA. In Supplementary Figure 6 (c), the band contrast image is constructed from all EBSD patterns. Dark areas in the images indicate regions without an observable Kikuchi pattern (i.e., these could not be indexed to the Sb_2_Te_3_ crystal structure), and bright gray regions show where the clear crystalline pattern was detected. Gray and black regions, therefore, indicate the crystalline and amorphous states, respectively. All of the irradiated surfaces in this regime of fluences were amorphous. The phase identification from the EBSD mapping is shown in Supplementary Figure 6 (d), where crystalline regions of Sb_2_Te_3_ appear as blue. The complementary AFM and EDS images show that surface amorphization occurs well before the onset of sputtering.


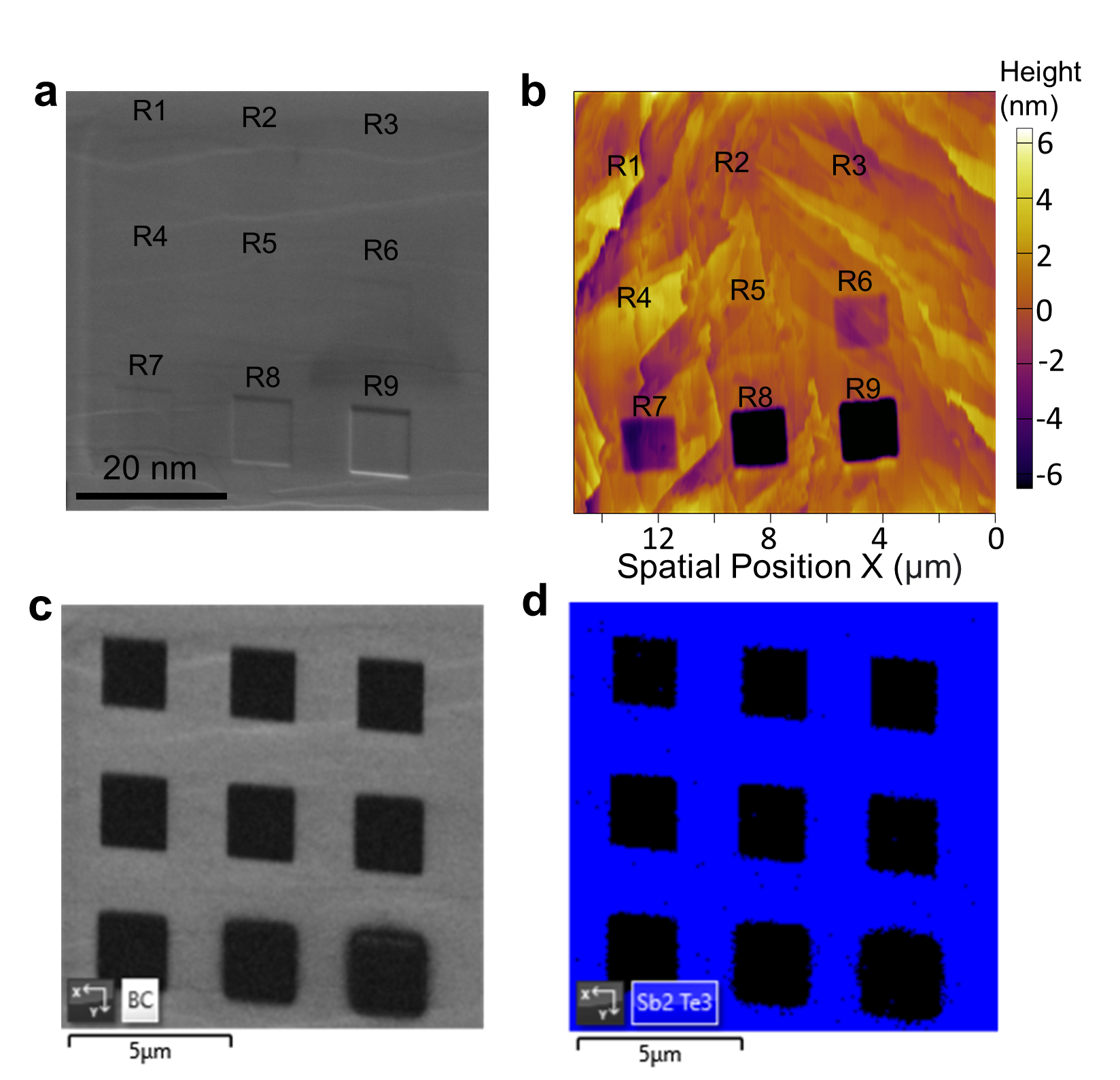


Supplementary Figure 6. (a) Secondary electron image of the 3×3 grid of irradiated regions labelled R1 – R9. Only the higher fluence regions (R7-R9) show topography due to sputtering effects. (b) AFM topography image labelled with the matching pattern ID (Note: the scan direction and imaging angle is different in the SEM/AFM images, as EBSD measurements require using an inclined surface). (c) Band contrast image obtained through an EBSD scan on the same region of interest, where amorphous regions appear black. (d) Phase color image showing the crystalline Sb_2_Te_3_ in blue and the non-indexable regions (amorphous) as black.

Supplementary Table 1. Identity of irradiated regions in Supplementary Figure 6, with corresponding ion fluences irradiated within the microscope at 8 keV (C/µm^2^) and conversion to the standard unit (ions/cm^2^).

| ID | C/µm^2^ | ions/cm^2^ |
| --- | --- | --- |
| R1 | 6×10^-14^ | 3.74×10^13^ |
| R2 | 1.2×10^-13^ | 7.49×10^13^ |
| R3 | 3×10^-13^ | 1.87×10^14^ |
| R4 | 6×10^-13^ | 3.74×10^14^ |
| R5 | 1.1×10^-12^ | 6.87×10^14^ |
| R6 | 2.3×10^-12^ | 1.44×10^15^ |
| R7 | 3.6×10^-12^ | 2.25×10^15^ |
| R8 | 9×10^-12^ | 5.62×10^15^ |
| R9 | 1.8×10^-11^ | 1.12×10^16^ |

## Patterning of circular and dot geometries

Systematic investigations were carried out to further investigate disorder profiles close to the crystal-to-amorphous phase transition and the prospects for geometric patterning of non-trivial shapes. Sb_2_Te_3_ surfaces were irradiated using a series of lower ion beam fluences in different geometries. The table below (Supplementary Table 2) reports the pattern sizes and corresponding ion fluence received for each pattern.

*Supplementary Table 2. Pattern ID and size with corresponding ion fluence irradiated within the FIB microscope (C/µm^2^) at 8 keV and conversion to the standard unit (ions/cm^2^).*


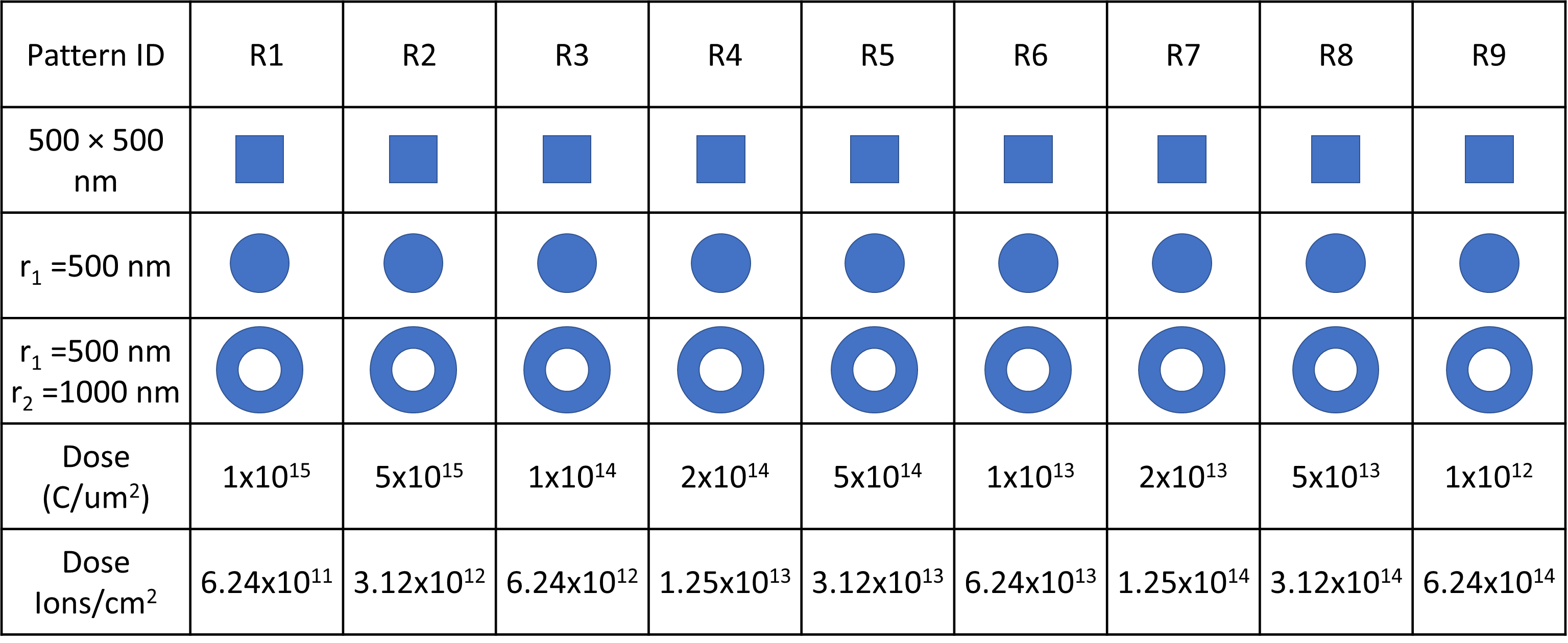


Supplementary Figure 7 (a) shows the electron image for the Sb_2_Te_3_ surface irradiated with the ion beam patterns described in the Supplementary Table 2. Supplementary Figure 7 (b) and (c) show the matching band contrast and phase color images. This indicates that the crystalline-amorphous transition of the Sb_2_Te_3_ surface begins to appear for patterns in column R5 (i.e. the critical fluence is ~ 3.12×10^13^ ions/cm^2^ for 8 keV Ga^+^). Supplementary Figure 7 (d) and (e) show the band contrast and phase color images of high-magnification EBSD scans for rectangular patterns R2-R5. Supplementary Figure 7 (f) shows the corresponding Kikuchi patterns of R3-R5. The Kikuchi bands start to disappear for the pattern R5, which is a signature of the amorphous transition. A further high magnification EBSD scan for ring-shaped patterns R6-R9 is shown in Supplementary Figure 7 (g) and (h) and indicate that any pattern which received Ga^+^ ion fluence greater 3.12×10^13^ ions/cm^2^ (5×10^-14^ C/µm^2^ ) was amorphized, for the 8 keV beam. Clear circular, crystalline dots can be formed, surrounded by an amorphous boundary with this patterning method. For much higher fluences, it is clear the damage spreads out further and the amorphous region gradually expands as shown by comparing the ring structures in R8 and R9 (Supplementary Figure 7 (g-h)). This study not only helps determine the critical fluence required to amorphize Sb_2_Te_3_ crystals; it also illustrates the possibilities for precisely “writing” lithographic patterns on the Sb_2_Te_3_ surface at lower doses.


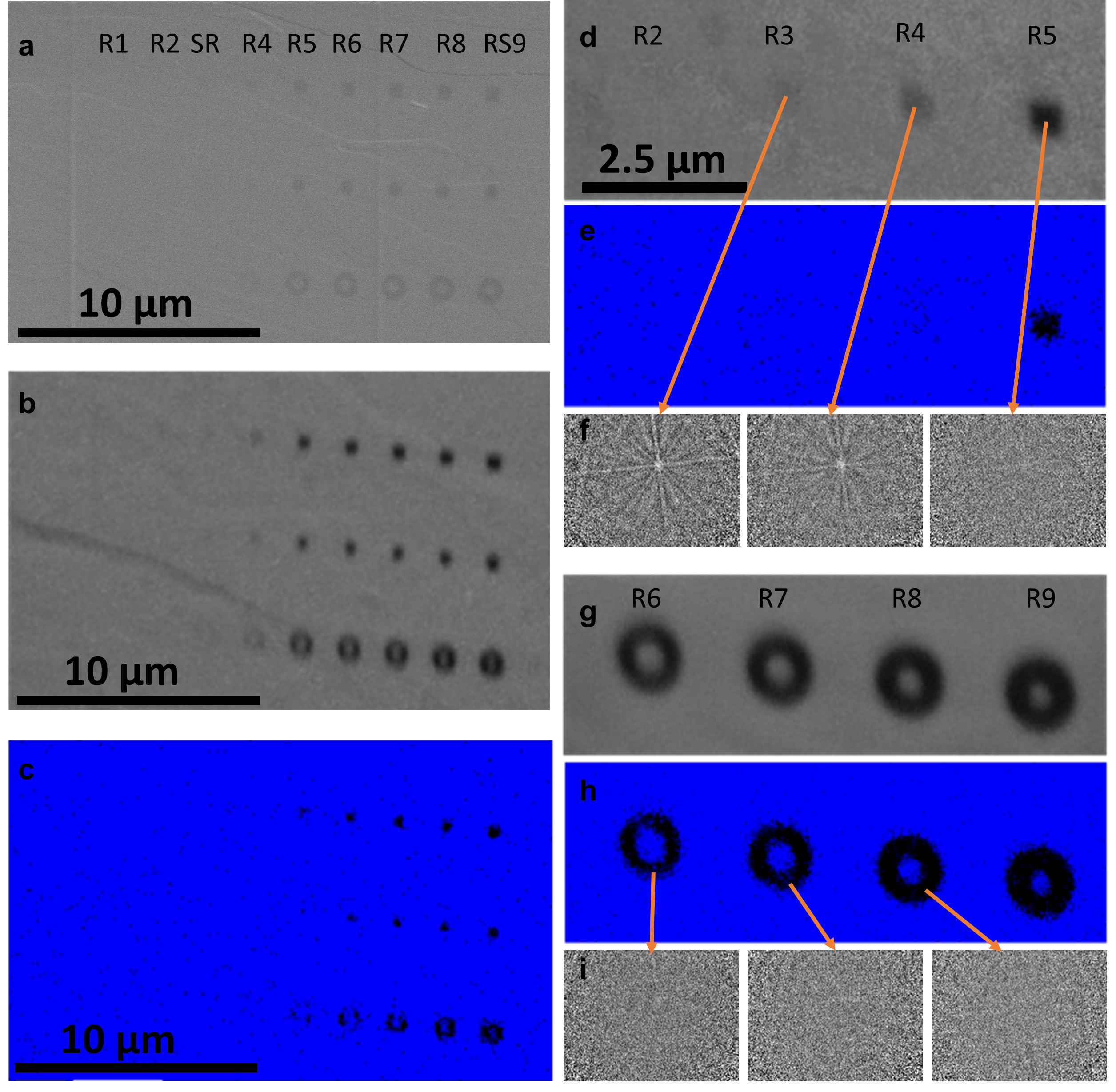


Supplementary Figure 7. (a) Scanning electron image with each pattern ID labelled on it, and (b) Band contrast image from EBSD scan. (c) Phase color image. (d) High-magnification band contrast image. (e) Corresponding phase color image. (f) Kikuchi patterns taken at R3, R4, and R5. (g) High-magnification band contrast image of the patterns with higher Ga irradiation dose. (h) Corresponding phase color image. (i) Kikuchi patterns within patterns R6, R7, and R8.

# Additional CAFM characterization

This section presents additional IV and line-profile measurements performed using a conductive atomic force microscopy (CAFM). Electrical characterization of ion-beam patterns was performed by a conductive atomic force microscopy using a commercial scanning probe microscope (Cyper S, Asylum Research, US). Conductive probes with Pt coating were used for both current mapping and current-voltage (I-V) measurements under a contact mode.

## Line profiles through a region patterned using high Ga fluences


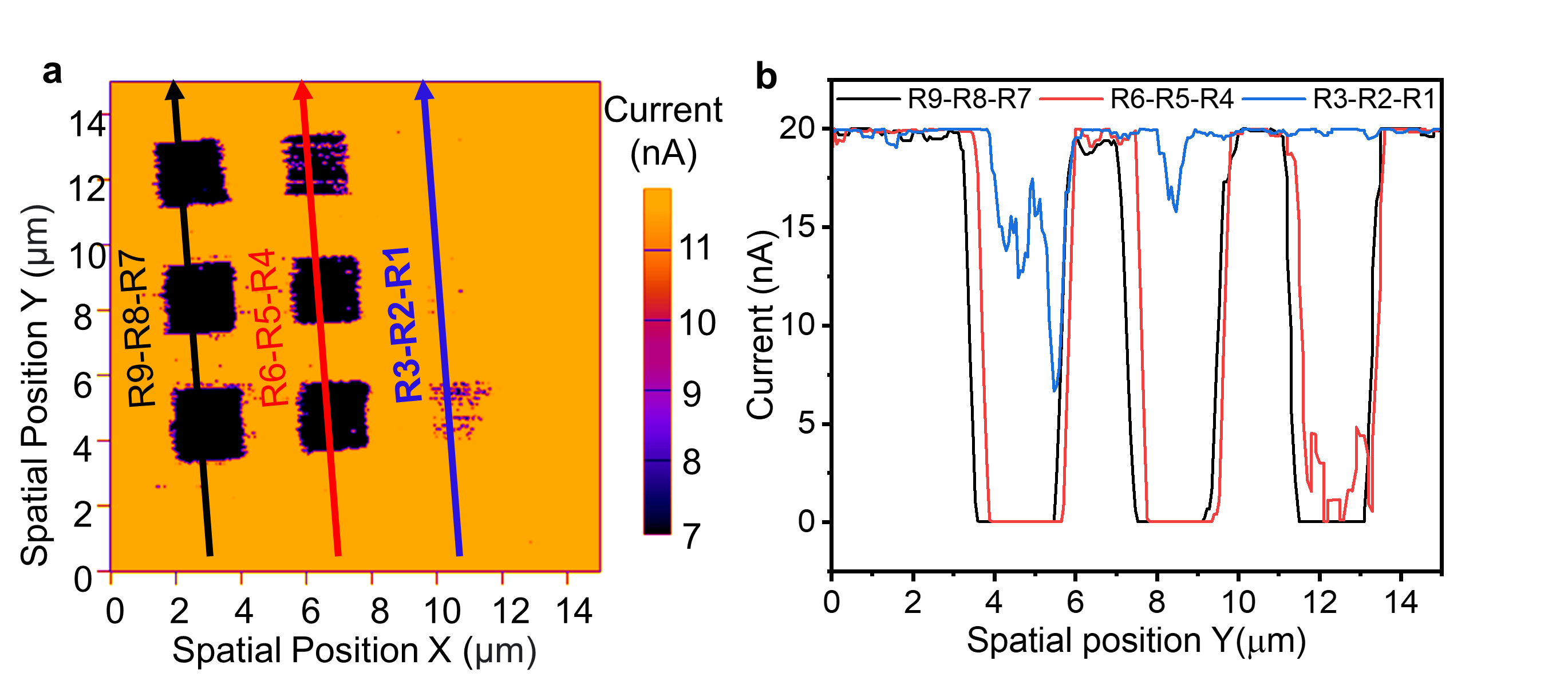


Supplementary Figure 8. (a) Line profiles through the array of irradiated grid squares (see Supplementary Figure 6 (a) and Supplementary Table 1 for details) for cAFM measurement at a scanning bias of 1V. (b) Current response along the line profile scans in (a) showing that the current decreases to zero within the most heavily irradiated grid squares (R4 – R9).

## I-V characterization of the lateral boundaries for regions patterned with low fluences

Supplementary Figure 9 (a) shows the I-V characteristics of several patterned regions, similar to the region shown in (b). The areas exposed to Ga have a significantly different I-V from the boundary and the unirradiated regions. Supplementary Figure 9 (b) CAFM image shows representative points where I-V scans were performed. We note that, while all of the scans over sufficiently irradiated regions show a large, consistent decrease in the current at the centre of A-V 2D boundaries from nanoamps to picoamps, the currents of the “edge” states at the A-C boundary only give contrast at specific bias voltages, and the current contrast is small (in the 50-100 picoamp range). This is near the sensitivity limits of the instrument. Concerning the anisotropic effect on the pattern edge conductivity for the “1D” C-A boundaries: this is due to the nature of conductive atomic force microscopy (CAFM), where the conductive tip was scanned along the X-axis (leftwards and rightwards). At the edges of the pattern where there are stages/steps, the contact area of the tip with the sample may vary slightly (with a higher or lower contact area depending on the surface condition). This causes the observed anisotropy around the pattern. To demonstrate this, in Supplementary Section 3.4, the samples were rotated, and scans were performed in various directions. Furthermore, to confirm the observed high edge conductivity from CAFM is real, we have also performed multiple point I-V measurements on several different irradiated areas and boundaries and compared these with neighboring crystalline areas. The results in Supplementary Figure 9 (c)-(e) confirm that the high conductivity from edges is reproducible, and the central amorphous areas of the pattern (at A-V surfaces) show a much lower current than both A-C edges and C-V substrate boundaries.


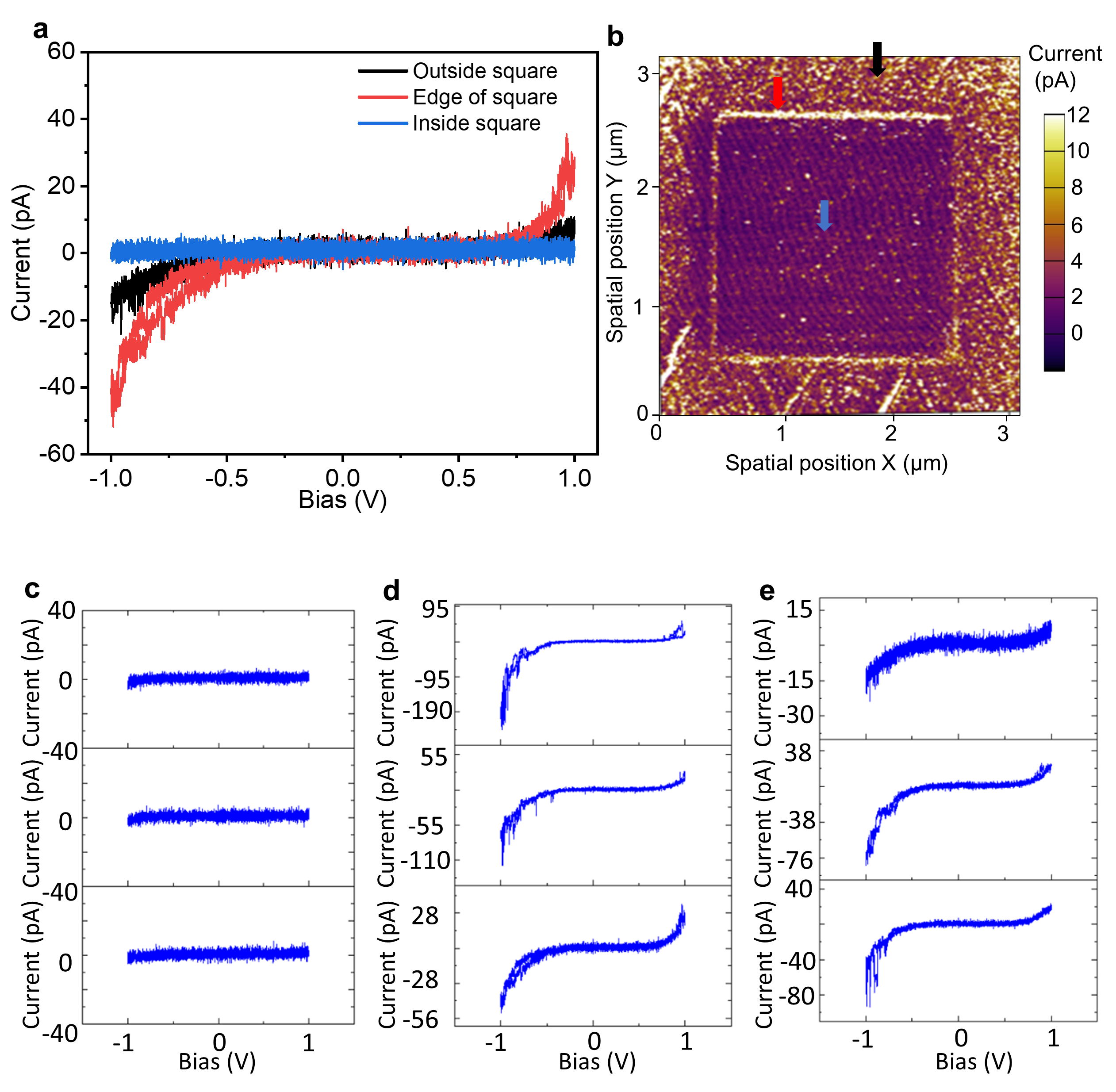


*Supplementary Figure 9. I-V characteristics. (a) Comparison of an ion-beam irradiated area (inside the midpoint of the square for an A-V boundary), with the A-C boundary (edges of the square), and unirradiated area at the crystallinity C-V boundary (outside the square). (b) CAFM image with colored arrow labels indicate the irradiated area, boundary, and crystalline area where I-V scans have been performed. (c-e) A series of repeated I-V scans at multiple patterned areas, edges, and crystalline regions to confirm the reliability of the measurements.*

## Line profile showing edge conductivity

A line scan profile on the CAFM image in Supplementary Figure 10 shows a clear current spike at both boundaries and terraces of the crystal, indicating that the high conductivity possibly originates from edge states.


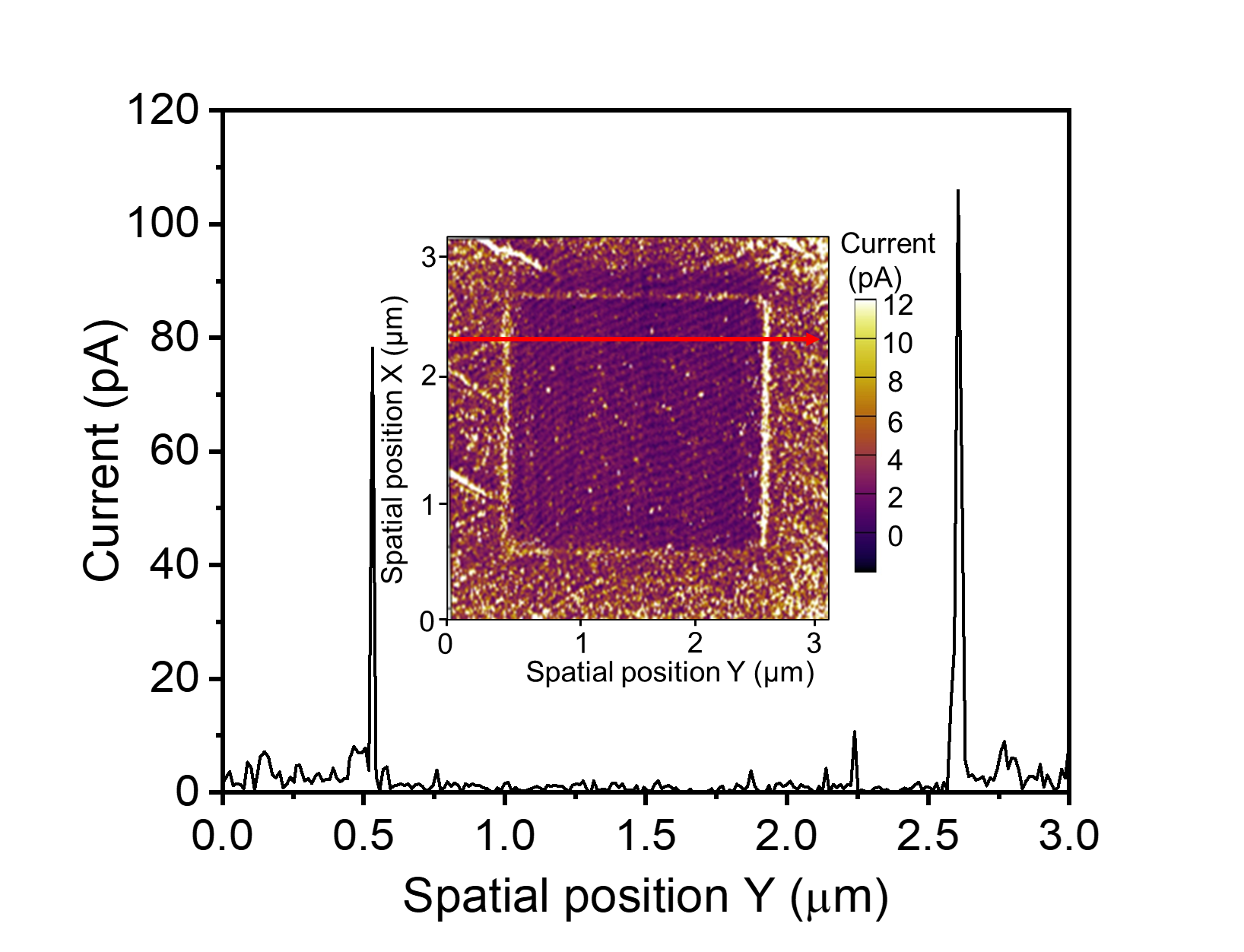


*Supplementary Figure 10. The current line scan profile shows the high conductivity at the edges at the boundary between ion-beam irradiated and unirradiated areas. (Note: the CAFM image is rotated so that the line profile direction matches the x-axis of the figure.)*

## Characterizing the anisotropy in the CAFM scans

Supplementary Figure 11 (a) and (b) CAFM images are collected from the same pattern, but the scanning angle has been deliberately rotated by 90 degrees (as indicated by the tip position and the arrows showing the fast/slow scanning directions). The numbers inside the figures label the same edges before and after the rotation. For either scan, the edges of the pattern show higher conductivity and irradiated areas show relatively lower conductivity.

Supplementary Figure 11 (a), edges 1 and 3 show relatively higher current than edges 2 and 4; however, in Supplementary Figure 11 (b), edge 1 and 4 both show higher conductivity. Please note the scale of the current value is quite small (within 15 pA) and probe-sample contact issues (as mentioned above) explain the observed anisotropy in the edges; however, the existence of the edge currents is well-supported by the data.

*
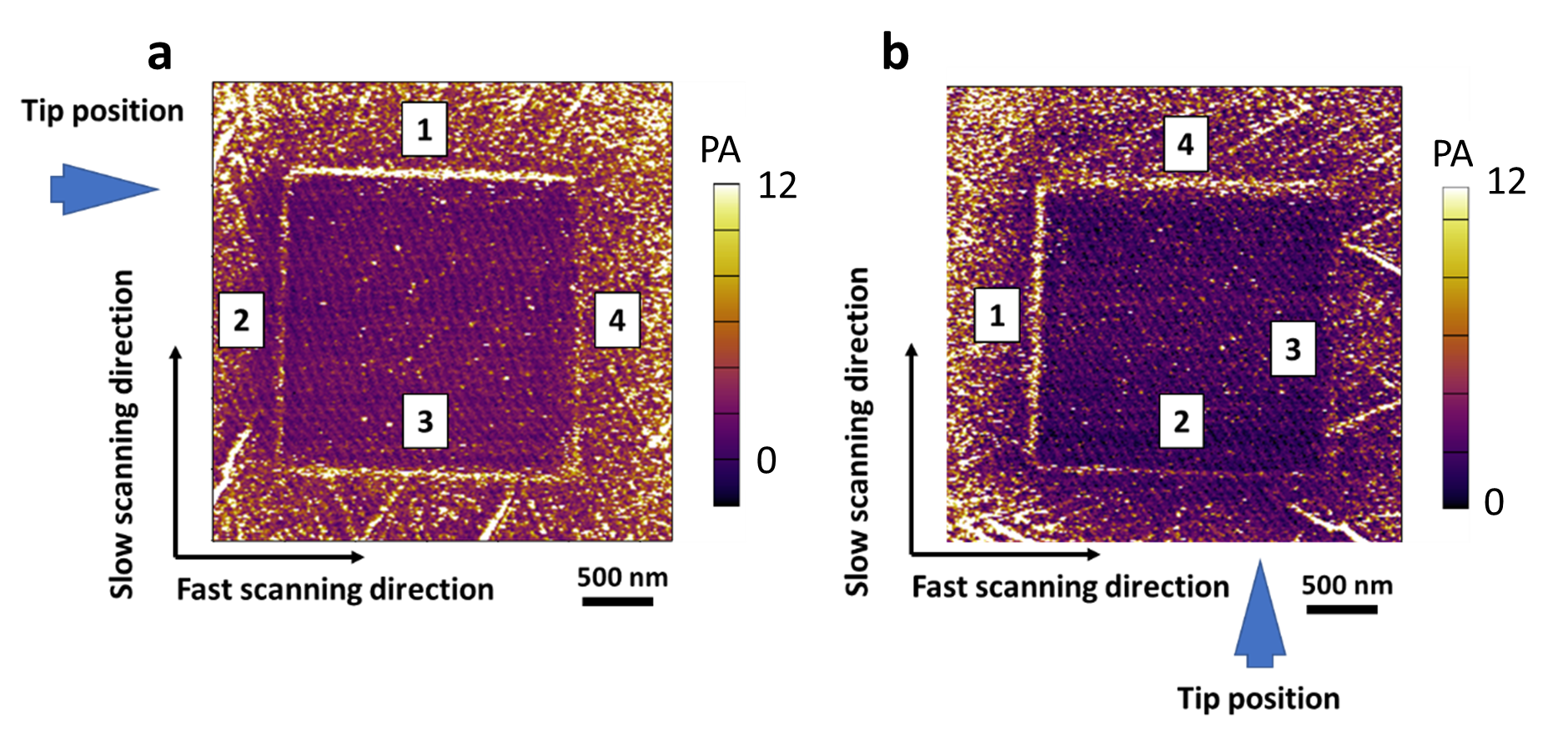
*

*Supplementary Figure 11. Current mapping using 1V DC bias for an individual irradiated box. (a) CAFM scans with a fast-scanning direction parallel to the probe. (b) CAFM scans with a fast scanning direction perpendicular to the probe.*

Supplementary Figure 12 shows a triangular-shaped logo that includes several edge lines at different angles to the scan direction. All the edges show an observable, higher conductivity (including the small circles inside the pattern), while the central parts of the irradiated pattern show lower conductivity. Our CAFM results collectively confirm the high conductivity at the boundary, although future work is needed to provide quantitative measurements.


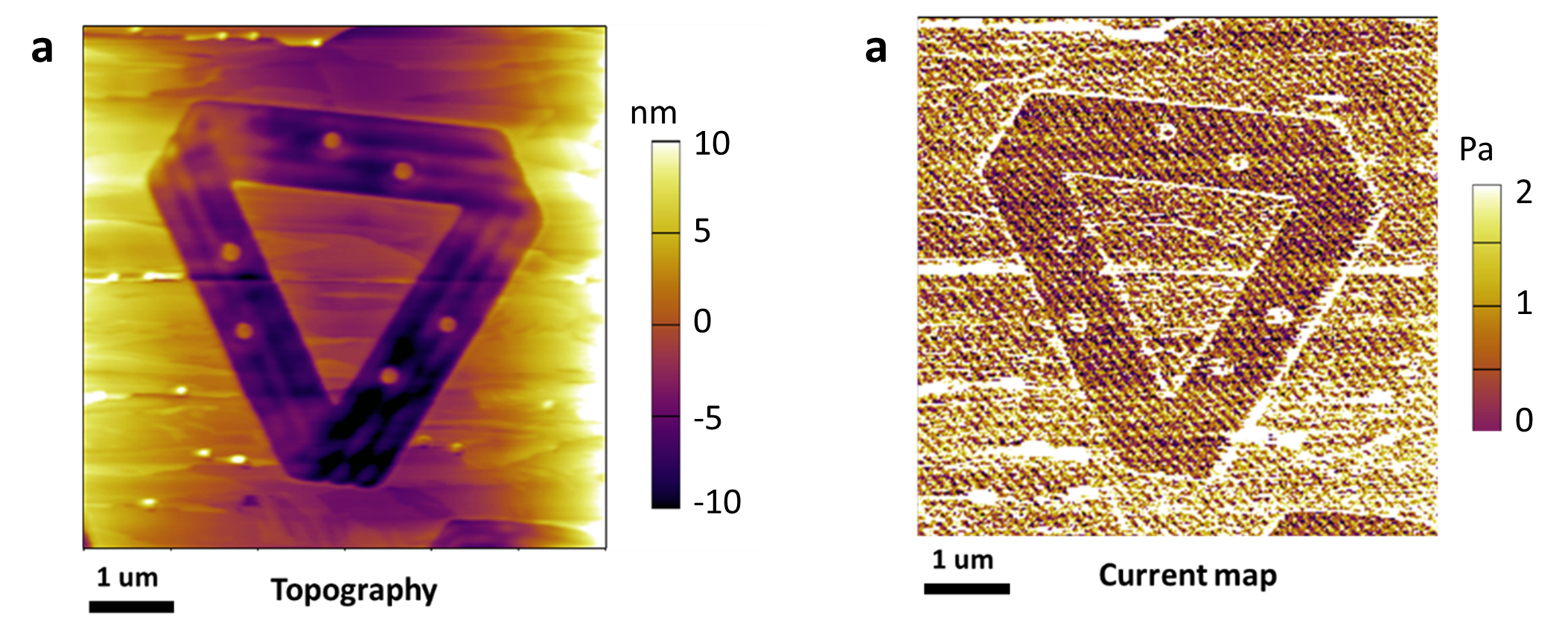


*Supplementary Figure 12. CAFM images of a patterned logo with edges at different angles to the scan direction. (a) Topography. (b) Current mapping of an individual irradiated FLEET logo pattern.*

# Details of transport measurements in the focused ion beam and thin film devices

## Details on the focused-ion beam device preparation methodology

To enable four-probe resistivity measurements on crystalline devices, a method was developed to prepare devices with minimal exposure to the focused ion beam, taking advantage of the van-der Waals (flaky) nature of Sb_2_Te_3_, and using the nanomanipulator. Supplementary Figure 13 illustrates the main steps. The details are summarized below:

1. First, we exfoliate the Sb_2_Te_3_ single-crystal surface with adhesive tape to liberate thin flakes on the surface. Under SEM imaging mode, we search for the flakes of desired thickness.
2. At low ion-beam voltage and current (8-16 KeV, <80 pA), we cut the desired size of lift-out, having welded a nano manipulator to one end of Sb_2_Te_3_ rectangular lift out. The process aims to avoid, as much as possible, exposing the surface of the lift-out to the ion-beam. Imaging of the procedure involves taking low current snapshots in ion-beam imaging mode at 8 KeV, <25 pA. Thus, the majority of surface of the flake lift out is unirradiated although one edge is slightly irradiated by a negligible amount of the Ga ion beam.
3. The rectangular lift-out is carefully placed on a set of pre-made contacts on a commercial chip. If any ion beam imaging is performed, this is done at very low voltage and current for a short time (<=8 keV, =<25 pa) to ensure the surface of the lift-out remains crystalline.
4. Pt deposition in the FIB is used to fabricate four-probe and Hall measurement contacts which link the gold pads on the chip to the surface of the crystal.
5. The crystallinity of the main areas of the device is confirmed using electron back-scatter diffraction (see Supplementary Figure 14).


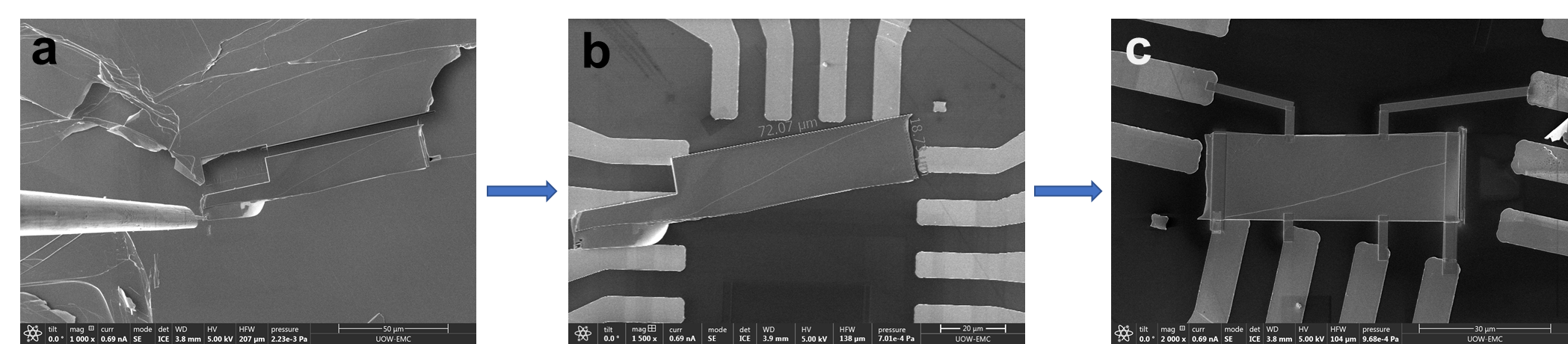


*Supplementary Figure 13. Device fabrication procedure for the crystalline FIB devices. (a) A thin exfoliated flake region is selected, and a single cut is made to remove a bar-like section and weld it to a nanomanipulator. (b) The flake is placed on prefabricated gold contacts. (c) Platinum contacts are deposited, linking the gold contacts to the surface of the Sb_2_Te_3_ bar (thickness=500-600 nm).*

## *Crystallinity of an as-prepared device*

The Supplementary Figure 14 a shows the electron image with superimposed EDS mapping after the fabrication of four-probe and Hall resistivity contacts. Supplementary Figure 14 b shows the EDS mapping (together with an electron image in the top panel) of the Sb, Te, Si, Pt, and Ga elements. The Si X-ray signals arise from the underlying Si chip, which supports the rectangular topological insulator device. The rectangular device thickness is in the range of 500-600 nm, and X-ray spectra originate not only from the Sb_2_Te_3_ device, but also from the micron deep regions from the underlying Si chip. Contacts were fabricated with ion-beam deposited Pt. Supplementary Figure 14 c shows the EBSD map (the band contrast image is at the top) and Supplementary Figure 14 e is an inverse pole figure coloring key for the EBSD map. Supplementary Figure 15 b and c show the electron backscattered Kikuchi patterns (EBSP) and corresponding simulated solutions in the Aztec software taken from the points B and C respectively on Supplementary Figure 15 a. The presence of clear Kikuchi patterns indicates that the detectable surface of the Sb_2_Te_3_ rectangular device is still crystalline after the contact fabrication steps. Supplementary Figure 15 d shows the 3D phase view of the hexagonal crystal structure of Sb_2_Te_3_, which is generated in the Aztec software.


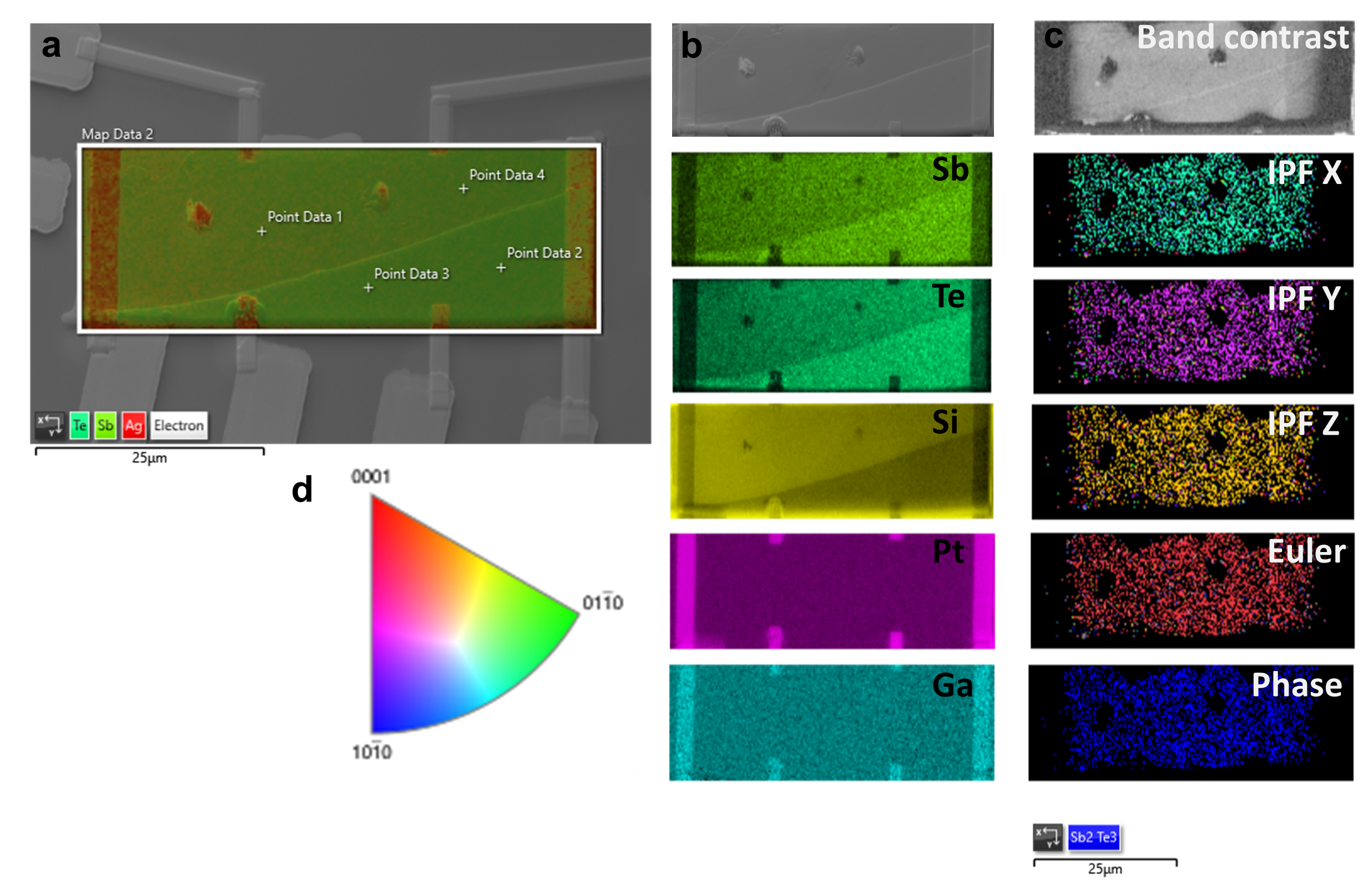


Supplementary Figure 14. EDS and EBSD characterization of the rectangular lift-out on a chip shows that the device is crystalline. (a) EDS layered image. (b) EDS elemental map taken from the white box in (a) (SE image at the top), and corresponding (c) EBSD and crystal orientation map with (d) inverse pole figure (IPF) coloring key.


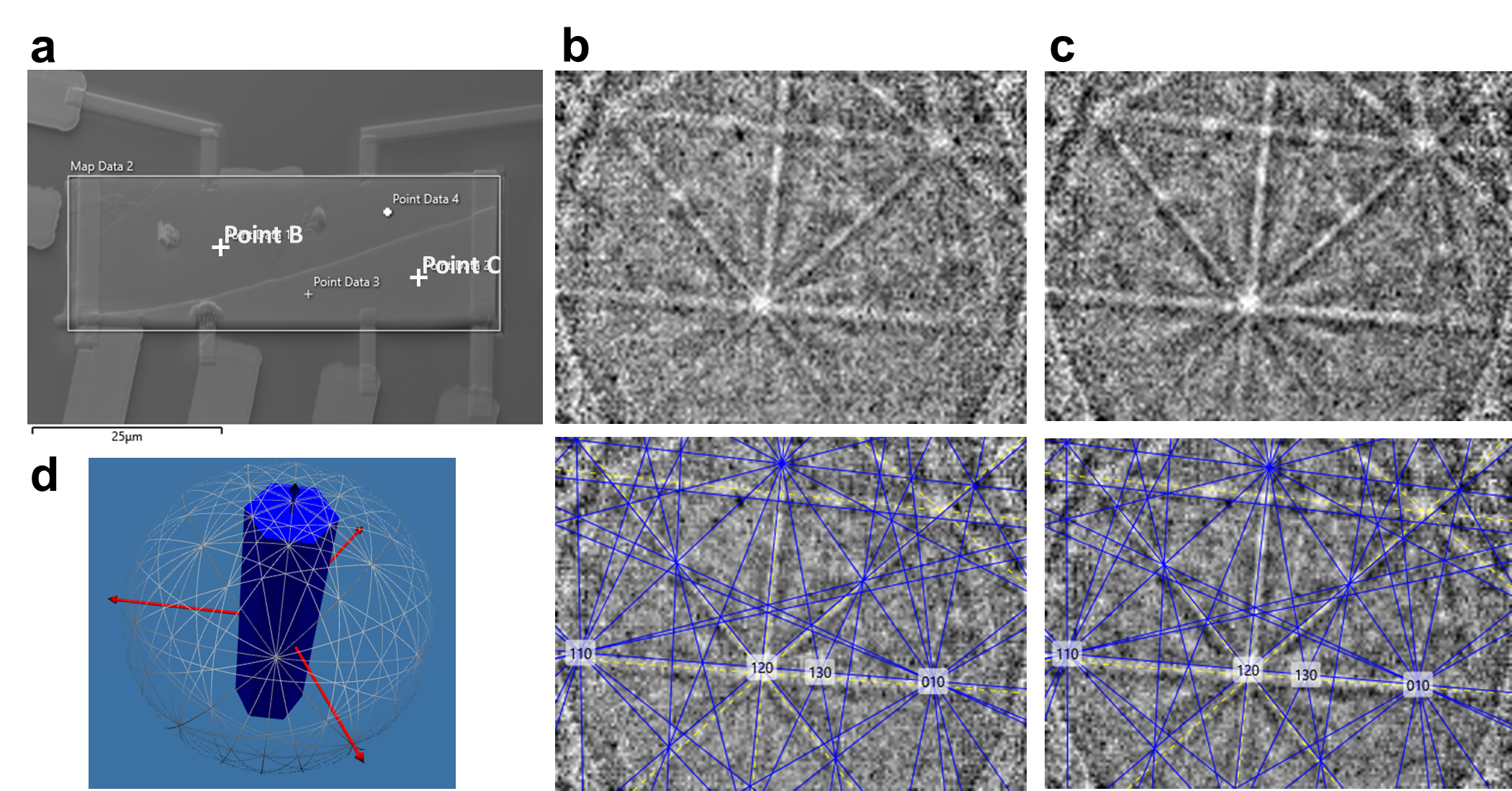


Supplementary Figure 15. Representative Kikuchi patterns for sections of the device showing it to be crystalline. (b) and (c) Electron back-scattered Kikuchi Patterns (EBSD) (with simulated solutions generated within the Aztec software) from point B and point C on the SE image in (a). (d) The 3D phase view of the unit cell shows the orientation is identical for points B and C, indexed to the hexagonal crystal structure of Sb_2_Te_3_.

## Shubnikov-de-Haas oscillations of the as-prepared crystalline device

To confirm that the device exhibits the quantum oscillations, characteristic of the bulk crystalline Sb_2_Te_3_ Fermi surface, magneto-transport experiments were performed on the device shown in Supplementary Figure 15. The temperature-dependent resistivity of the device is shown in Supplementary Figure 16 a, indicating a metallic-like resistance characteristic of Sb_2_Te_3_ where the Fermi level is positioned in the bulk valence band. The magnetoresistance measurements are shown in Supplementary Figure 16 b, from which the oscillatory component is extracted in Figure 16 c. The Fourier transforms of the oscillations shows that the main component is very similar to past reports for crystalline Sb_2_Te_3_^5^.


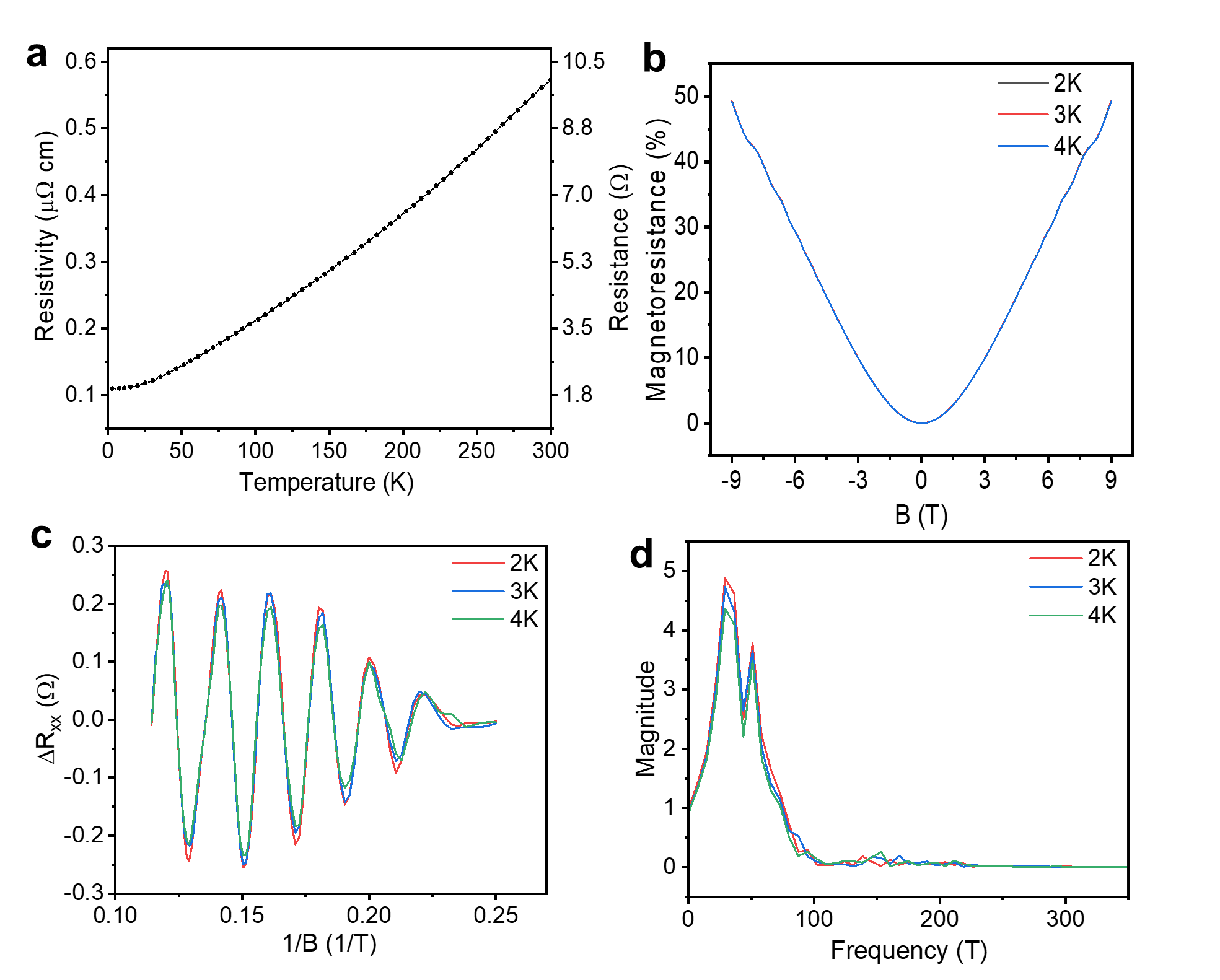


Supplementary Figure 16. Transport measurement of the FIB fabricated device. (a) RT measurement. (b) Magnetoresistance at 2, 3, and 4 K. (c) Shubnikov-de Hass (SdH) oscillation extracted by subtracting the background from the magnetoresistance measurement. (d) Fast Fourier transform (FFT) magnitude of the SdH oscillations measured at 2$-$4K.

## Effect of irradiation and post-annealing in the FIB-fabricated device

To investigate whether the amorphous transition can be reversed, an in-situ heating experiment was conducted as shown in Supplementary Figure 17. First, a bar similar in dimension to the devices was prepared on a heating element. The EBSD Euler map in Supplementary Figure 17 a shows that the bar is initially crystalline as it can be indexed to Sb_2_Te_3_. The elemental maps of each component are also illustrated in the EDS maps. To selectively amorphize a region of this bar, a high dose of 7 pA Ga, at 30 keV was used to irradiate an area of 5x3 µm in the center of the bar. The EBSD band contrast and Euler maps in Supplementary Figure 17 b show that this region becomes amorphous. The sample was then heated to 400°C and held for 0.5 hour in-situ. After heating, Supplementary Figure 17 c shows the amorphous region recrystallized, evident from the re-appearance of the electron back-scattering diffraction (Kikuchi) pattern shown in the band-contrast image.


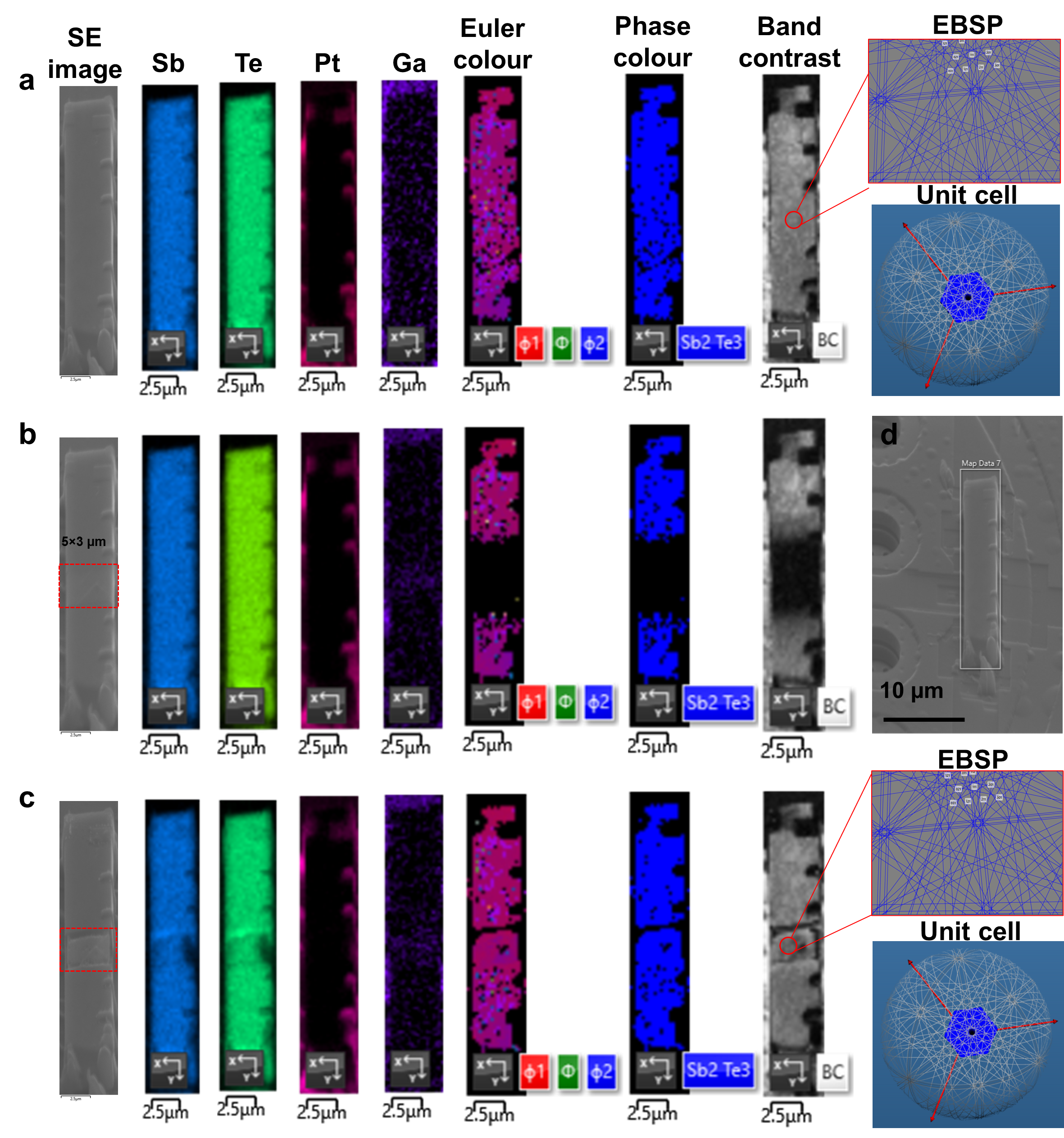


Supplementary Figure 17. In-situ characterization of a Sb_2_Te_3_ bar before irradiation, post-irradiation and post-heating. (a) EDS and EBSD scan of a rectangular lift-out section welded on an FEI heating chip before irradiation. The EBSD and unit cell image indicate the lift-out surface's crystalline nature. (b) EDS and EBSD scan after the 5x3 μm area (indicated in the red box on the SE image) was irradiated with a Ga ion beam. The EBSD scan highlights the amorphous nature of the irradiated area. (c) After in-situ heat treatment at 400 °C for 30 minutes, the EBSD images show the recrystallization of the Sb_2_Te_3_ region via the disappearance of the dark amorphous region in the band contrast images. Images were generated within the Aztec software.

## *Lumped-element model for the FIB fabricated devices*

When intentionally irradiating the rectangular lift-out device with Ga ions at 30 KeV, a surface amorphous layer of approximately t_s_=50 nm thick is formed. For lower incident energies (8 keV), the amorphous layer is approximately 20 nm thick (see Supplementary Figure 2). Based on the four-probe measurement configuration and a scenario where a FIB device has an amorphous surface layer, and a crystalline underlayer, we created a lumped element model as in Supplementary Figure 18. This introduces two parallel conduction channels for the amorphous surface layer (modelled by a resistor R_S_) and the crystalline underlayer (R_B_). As the contacts are made to the surface layer, any current which passes through the bulk must first pass through a series resistor that models the surface leakage resistance (R_L_). The total (net) resistance of the device can be approximately calculated with the Supplementary Equation (2) below:

$R_{T}=\frac{R_{S} \left( 2R_{L}+R_{B} \right)}{R_{S}+R_{B}+2R_{L}}$ Supplementary Equation (2)

where R_T_ is the total resistance, R_S_ is the surface resistance, R_B_ is the bulk resistance, and R_L_ is the leakage resistance. Based on the device measurements (see main manuscript Figure 2 (f)), the total room-temperature resistance of the device when the surface is crystalline is R_T_ = 22.23 Ω. After surface amorphization, R’_T_ = 89 Ω. From Supplementary Figure 19, we estimate the leakage resistivity of the amorphous Sb_2_Te_3_ is in the range of 1 × 10^6^ Ω-μm to 1 × 10^7^ Ω-μm. Using the device dimensions with l = 50 µm, w = 15 µm, t = 0.5 µm and t­_s_=50 nm, the value of R_S_ is 6.67 ×10^8^ Ω, and assuming that leakage occurs through the entire surface area (l×w) over length t_s_, R_L_=67-667 Ω. This gives the total resistance of the amorphized device of approximately 170–1370 Ω, which is in the same order as observed experimentally (88 Ω). We, infer that pinholes and imperfections in the nanoscale amorphous layer substantially decrease the leakage resistance. R_L_ is reduced to 26 Ω, to account for the observed total resistance of 88 Ω observed experimentally.


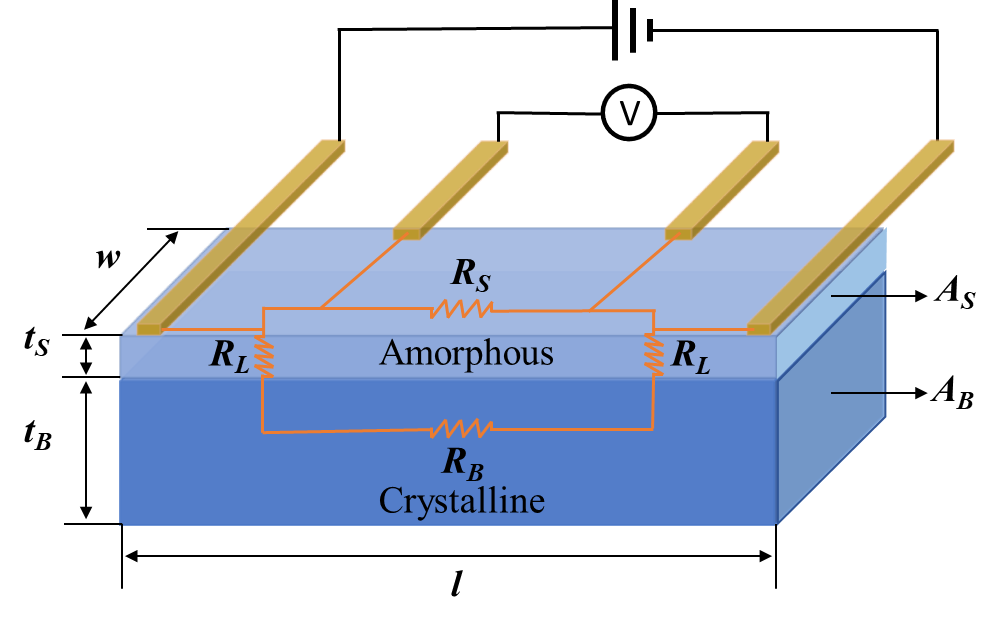


Supplementary Figure 18. Schematic illustration showing the configuration for the four-probe contact measurements and the corresponding lumped element model of a Ga-FIB device with an amorphous surface layer, and a parallel bulk conducting channel.

Supplementary Table 3. Total resistance calculation of the FIB device based on the lumped element model.

|  | Resistivity, ρ (Ω.µm) | Area, A (µm^2^) | Surface area | Resistance, R (Ω) |
| --- | --- | --- | --- | --- |
| Bulk (R_B)_ | 5 | 6.75 | *A_B_= w x t_B_* | 37 |
| Surface (R_S_) | 1 × 10^7^ | 0.75 | *A_S_= w x t_S_* | 6.67 ×10^8^ |
| Leakage (R_L_) | 1 × 10^6­^ -1 × 10^7^ | 750 | *A_L_= w x l_CONTACT_* | 67-667 |
| Total resistance (FIB device R_T_ = 22.23 Ω, R_T_’(Amorphous surface) = 89.45 Ω) | | | | 170 -1370 |

## Complementary thin film devices

The FIB devices shown in the previous section have an amorphous surface and a crystalline bulk region. This introduces parallel conduction as illustrated by the lumped element model described in section 4.5. In order to eliminate the bulk crystalline conduction channel and measure the intrinsic response of the amorphous Sb_2_Te_3_ itself, subsequent experiments utilized thin film Sb_2_Te_3_ which can be fully amorphized at the available Ga-ion beam energies. This allows for a clearer study of the intrinsic resistance of the amorphous phase, and the behavior of the A-V boundary.

Thin films were grown by molecular beam epitaxy on single crystal Al_2_O_3_ (001)-orientated substrates, with a total thickness between 5–30 nanometers. The lateral area of the samples was 10 × 10 mm^2^. The crystallinity was confirmed by their RHEED patterns, and XRD (see later). The resistivity of a crystalline film is shown in Supplementary Figure 19 a, exhibiting a metallic resistance with an upturn at low temperatures. This entire film was then amorphized using a broad-beam low-energy ion beam accelerator, with Ga ions at 30 keV and a fluence of 1 × 10^15^ ions/cm^2^_._ This fluence was selected as it is above the Ga ion-beam amorphization threshold of crystalline Sb_2_Te_3_ (see main text, Figure 2 (d)).

After irradiation, the resistance of the film increased by almost six orders of magnitude, as shown by Supplementary Figure 19 b, as compared to the resistance of the crystalline counterpart as shown in Supplementary Figure 19 a. Furthermore, the resistivity exhibited an exponential increase with decreasing temperature – a hallmark of a good insulator. Unfortunately, low-temperature measurements could not be performed because the resistance became too high to measure accurately with the available equipment. This is attributed to the excellent insulating properties of the Sb_2_Te_3_ amorphous phase, and the complete suppression of any surface conducting channels. The transition between the crystalline as-grown film to the amorphous-irradiated film was confirmed using cross-sectional TEM images as shown by Supplementary Figure 19 c and d. To further understand this transition, and provide accurate measurements of the film morphology, low angle X-ray diffraction, X-ray reflectometry and neutron reflectometry were performed of the film before and after irradiation. Measurements were performed on the Rigaku Smart Lab X-ray instrument and the SPATZ neutron reflectometer at the Australian Centre for Neutron Scattering (ACNS) at ANSTO. The X-ray data are shown in Supplementary Figure 20. The disappearance of the (003) Bragg peak shows that the entire film was transformed to the amorphous state by the irradiation step. The noticeable shift in the low-angle Kiessig fringes shows that the film thickness slightly increased by several nanometers after irradiation due to swelling. A similar conclusion is reached using the neutron reflectometry data in Supplementary Figure 21. These data were quantitively fitted to a model constructed using the REFNX software package to obtain the 1D scattering length density profiles. The larger Q-range in the X-ray data allows for a higher resolution structural solution, where the modulations from the Sb_2_Te_3_ quintuple layers can be resolved (giving rise to the Bragg peaks at ~0.6 Å^-1^). The neutron dataset probed a lower Q-range and was fitted to the continuum model. Both datasets show that the film swelled from an initial thickness of 28nm to 30 nm after irradiation. This is attributed to the lower mass density of the amorphous phase, which is also consistent with the lower scattering length density (SLD) values observed in the irradiated phases. From the neutron and X-ray contrast, the mass density of the amorphous phase can be uniquely calculated as: 6.1 g/cm^3,^ which is reduced relative to the crystalline phase (6.5 g/cm^3^).


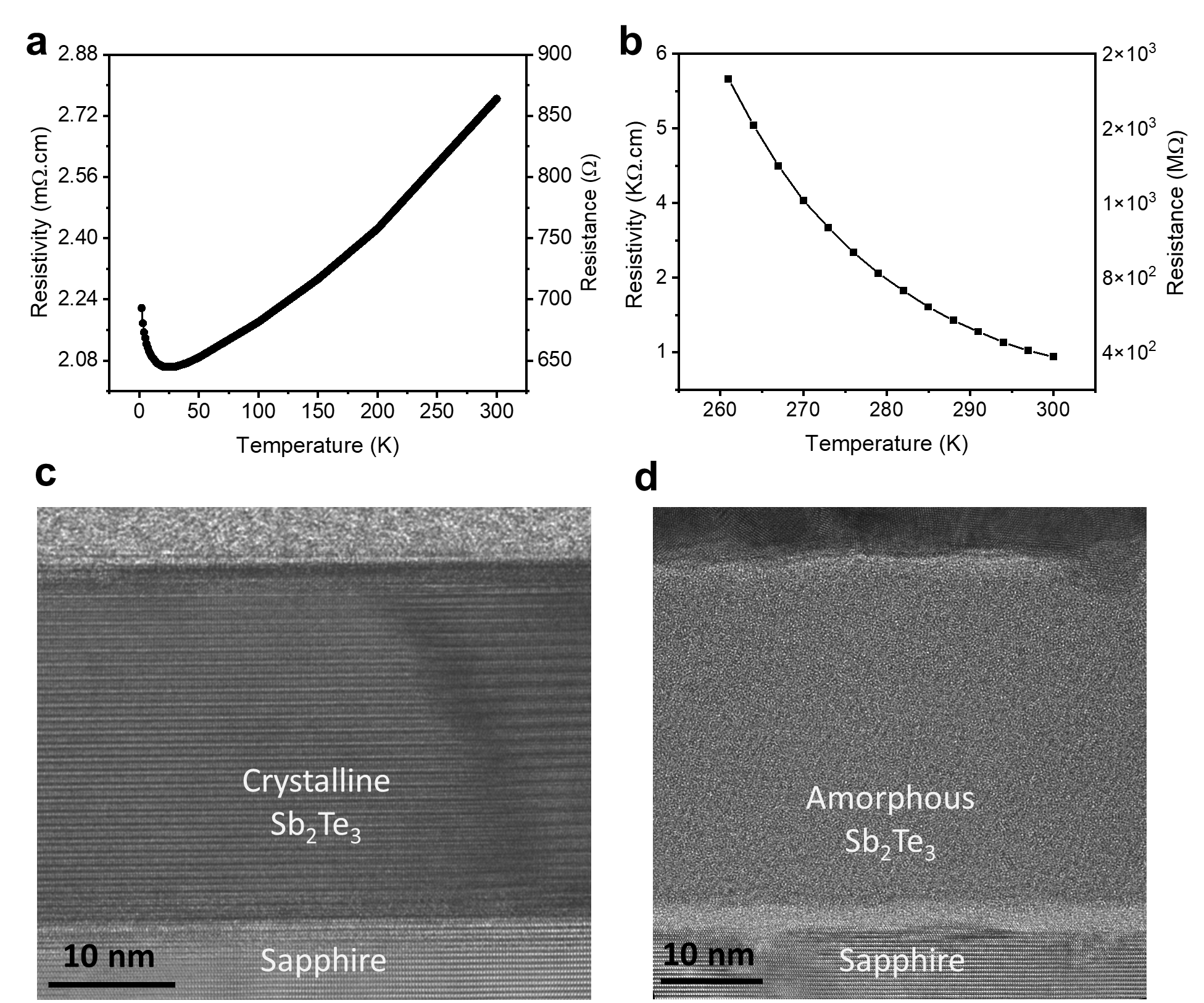


Supplementary Figure 19. Temperature-dependent resistivity of (a) the as-grown crystalline and (b) amorphized Sb_2_Te_3_ thin film grown by molecular beam epitaxy. (c) Cross-sectional TEM images of the starting film illustrating the clear lattice fringes from the crystalline order. D) Image of the same film after Ga-ion irradiation using a broad beam Low Energy Ion Implanter.


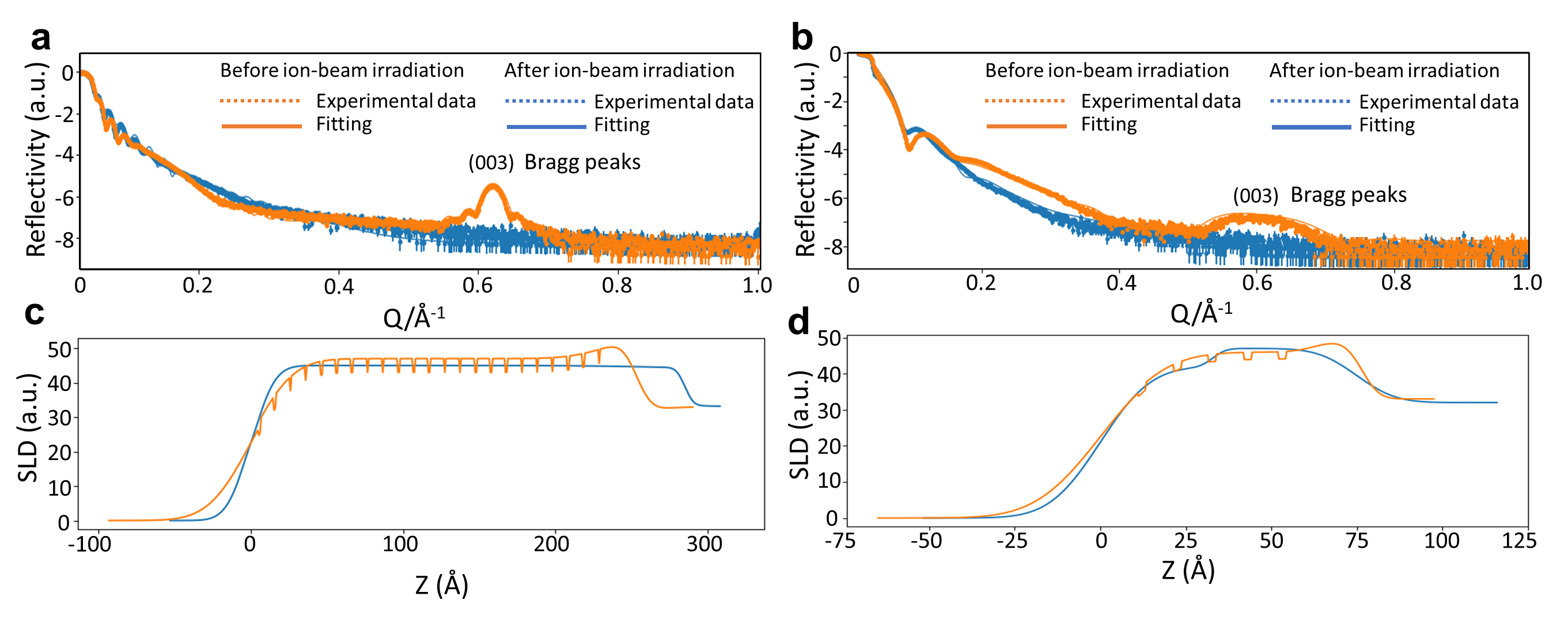


Supplementary Figure 20. X-ray reflectometry of (a) MBE-grown Sb_2_Te_3_ film before and after ion-beam irradiation. (a) ~28 nm thick film before irradiation (b) MBE-grown Sb_2_Te_3_ film (~10 nm) before and after ion-beam irradiation. (c) and (d) SLD profiles for (a) and (b), respectively.


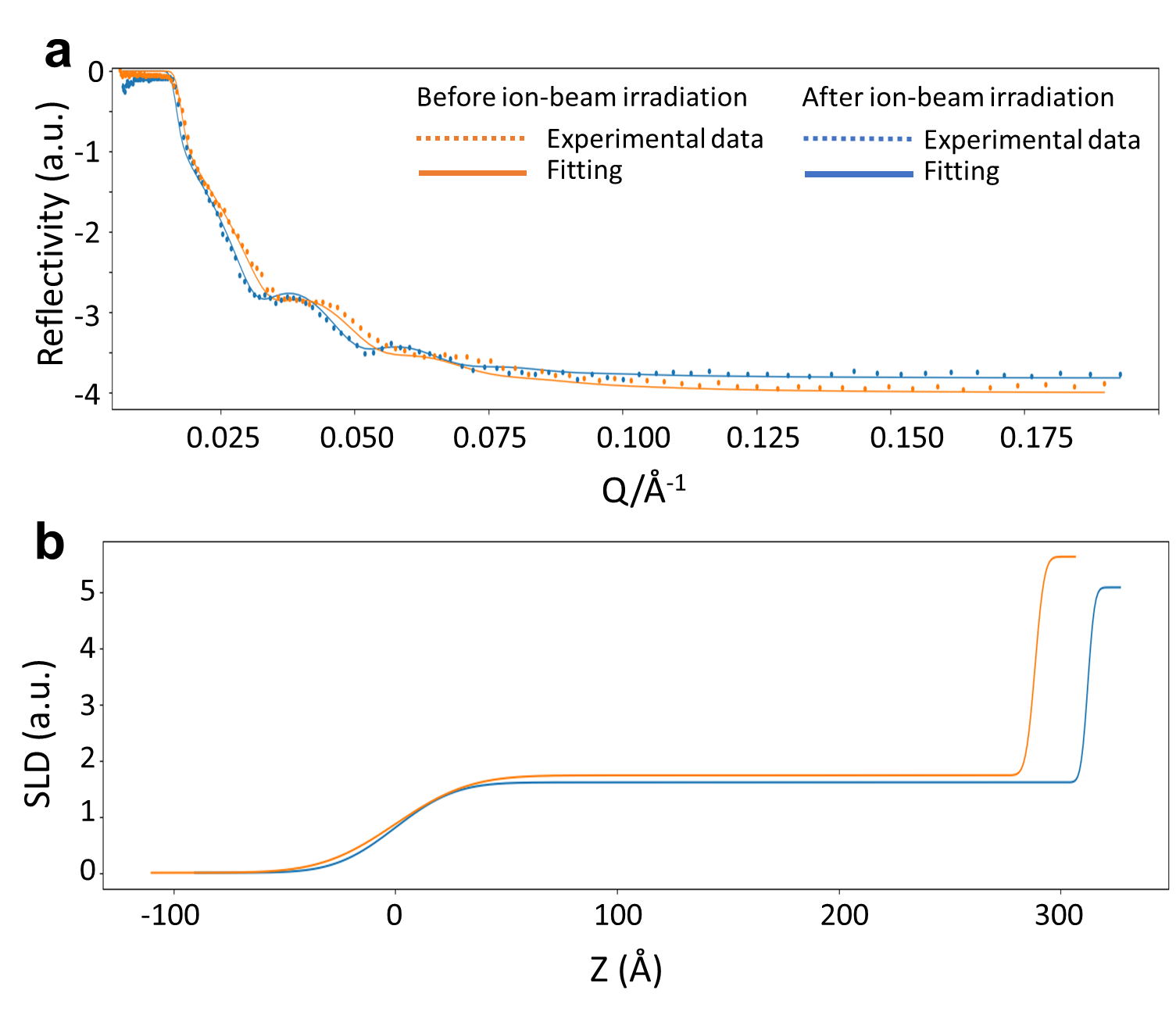


Supplementary Figure 21. (a) Neutron reflectometry of MBE-grown Sb_2_Te_3_ film (~30 nm) before and after ion-beam irradiation (b) Corresponding SLD profiles.

# Density functional theory calculations

## Finite temperature dynamics in Sb_2_Te_3_

This section presents a systematic set of *ab initio* molecular dynamics (AIMD) and DFT calculations which were used to construct the models presented in the main paper.

As a first step, it was necessary to test whether AIMD correctly described the finite-temperature vibrational dynamics of Sb_2_Te_3_, as this is central to the melt-quench simulation. Using a calculation at thermal equilibrium, the vibrational density of states of Sb_2_Te_3_ was calculated by Fourier transforming the velocity correlation functions from the AIMD trajectory, which is a standard approach. This was compared with experimental inelastic neutron spectroscopy measurements of the crystalline state at 77 K (Supplementary Figure 22). The distinctive phonon vibrational spectrum was found to be in the range between 1–5 THz (1–20 meV) in Sb_2_Te_3_, both in experiment and theory. The soft phonon modes are typical of heavy chalcogenide compounds, and the main peak positions are correctly predicted by AIMD. Peaks in the density of states appear at ~ 1.2 THz, ~ 1.9 THz and 3 and 4 THz both in the experiment and calculations, corresponding to the van-Hove singularities of the Sb_2_Te_3_ phonon band structure. The relative peak widths/intensities are correlated, and theory does not reproduce these with perfect accuracy. This is due to the relatively small supercell (4x4x1) available in AIMD, which means that phonon lifetimes (linewidths) are affected by finite-size effects. However, overall the energetics are well reproduced, providing confidence that melt-quench simulations will have a high degree of realism.

Supplementary Figure 22. Vibrational density of states calculated with ab initio molecular dynamics (shaded region) and measured with neutron spectroscopy (points and black line) taken from Ref. ^6^

## Simulating the melt-quench dynamics for glass formation

To generate the glassy version of Sb_2_Te_3_, and therefore simulate the ultimate effect of the ion beam, the melt-quench method was employed as described in the Methods section, to simulate the crystal → liquid → glass transition by heating a virtual crystal to 1600 K and cooling it to 100 K in 12–20 picoseconds.

As the melt-quench process is stochastic, it generates a different microscopic glass configuration after each simulation. To ensure that the results were robust, and not unique to just one specific atomic arrangement, the calculations were repeated fifteen times using various sized cells. Additional calculations were also performed, including the van der Waals corrections using the method developed by Grimme *et al*. (notated as D3) ^7^. Supplementary Figure 23 shows the calculated radial distribution function of the glass, compared over several runs and also plotted against previously published data. This shows that the structure is relatively reproducible. Supplementary Figure 24 shows the calculated electron density, and atomic arrangement, for a typical glass configuration.

Supplementary Figure 23. The pair distribution function of amorphous Sb_2_Te_3_ simulated by the melt-quench method and compared with two past references.


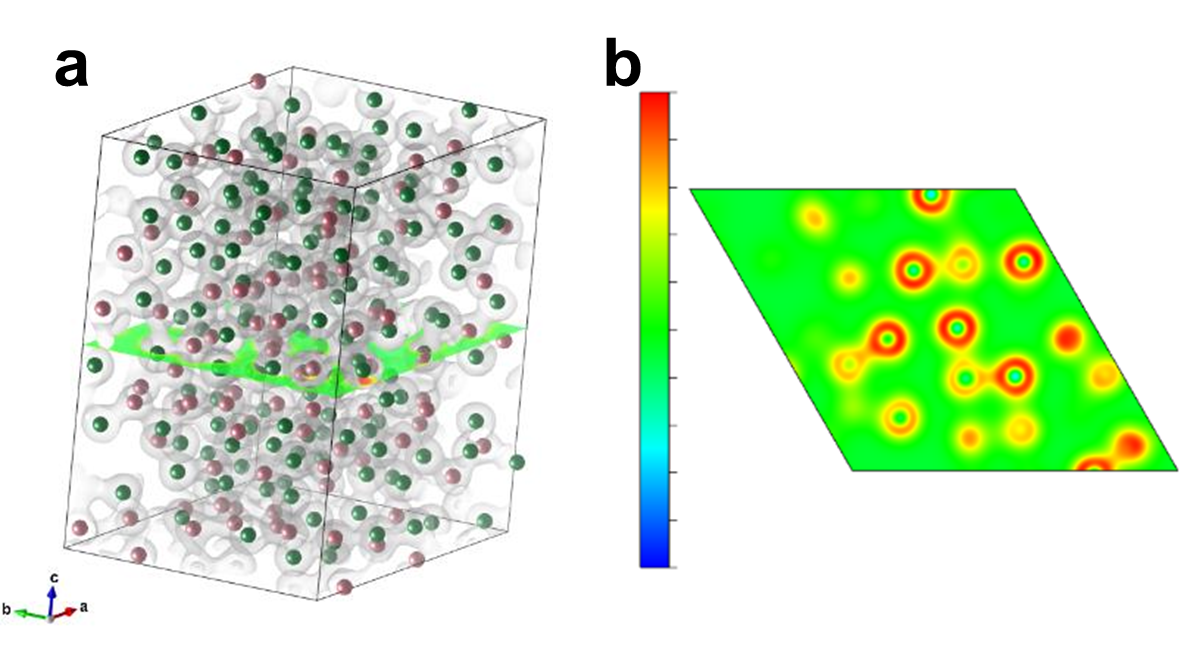


Supplementary Figure 24. Example electron density of amorphous Sb_2_Te_3_. (a) Electron density in a slab model. (b) A slice-plane through the electron density along the c axis of the slab.

## Electronic density of states and bandgap of the amorphous cell

The electronic density of states was calculated for the various glassy configurations. Calculations included the spin-orbit interaction. All simulations predicted a band-gap in the Sb_2_Te_3_, however the size of the gap varied from simulation to simulation as summarized in Supplementary Table 4.

*Supplementary Table 4. Summary of the band-gaps extracted from the electronic density of states of amorphous Sb_2_Te_3_.*

| Model Series | Number of atoms in cell | Number of structural configurations | vdW corrections included | Bulk band-gap (eV) | Band-gap standard deviation (eV) |
| --- | --- | --- | --- | --- | --- |
| I | 135 | 5 | No | 0.30 | 0.06 |
| II | 240 | 5 | No | 0.28 | 0.15 |
| III | 240 | 5 | Yes | 0.22 | 0.09 |

## Gallium impurity in the amorphous cell and amorphous surface

To model the electronic effect of the Ga impurity, additional calculations were performed using the same melt-quench methodology, except including a single gallium atom (modelling 0.5–1 atom % in the cell). The electron density of the supercell is shown in Supplementary Figure 25. The electronic density of states and the partial density of states is shown in Supplementary Figure 26.


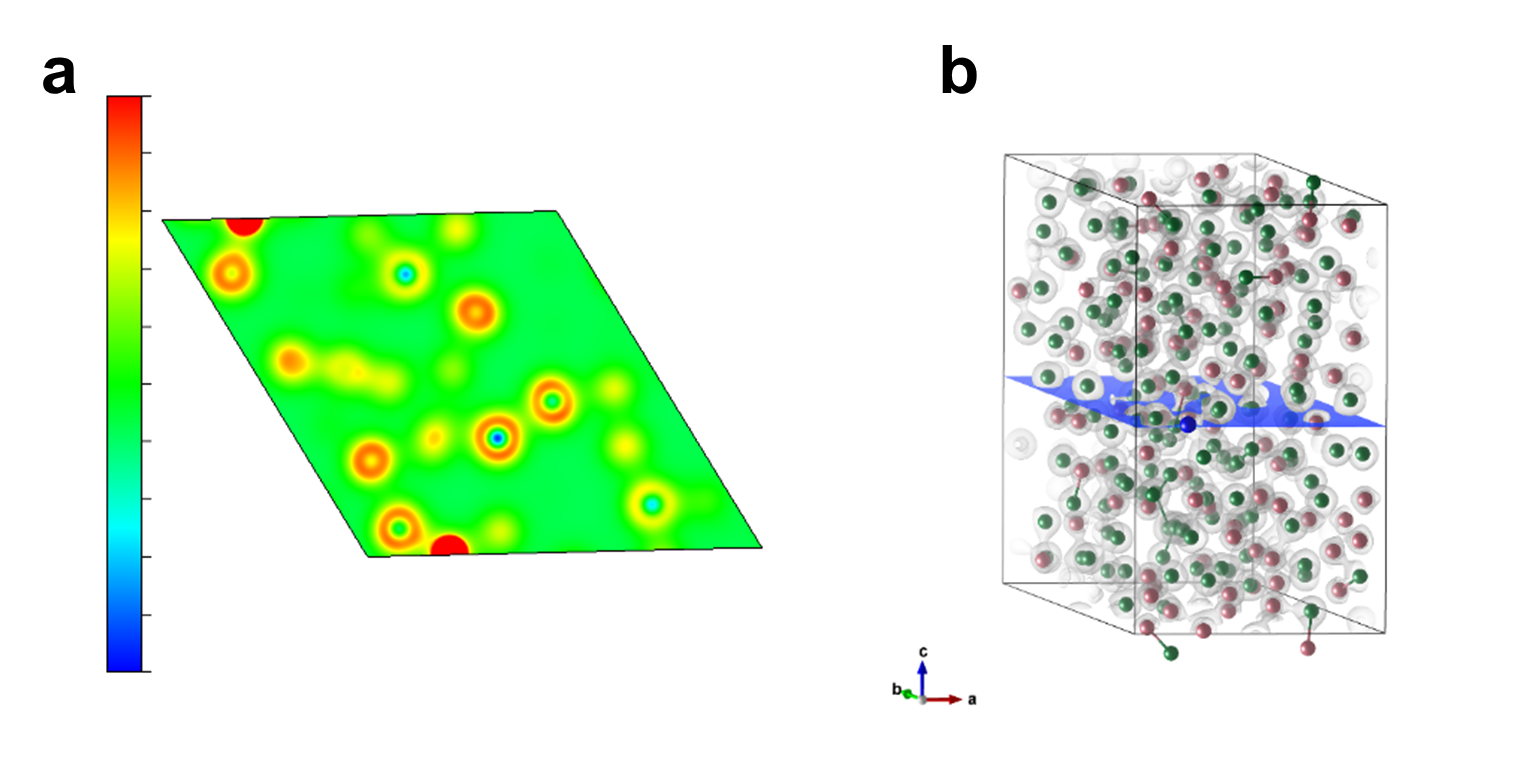


Supplementary Figure 25. Example electron density of amorphous Sb_2_Te_3_ with a Ga dopant. (a) A slice-plane through the electron density along the c axis of the slab in (b). (b) Electron density in a slab model.


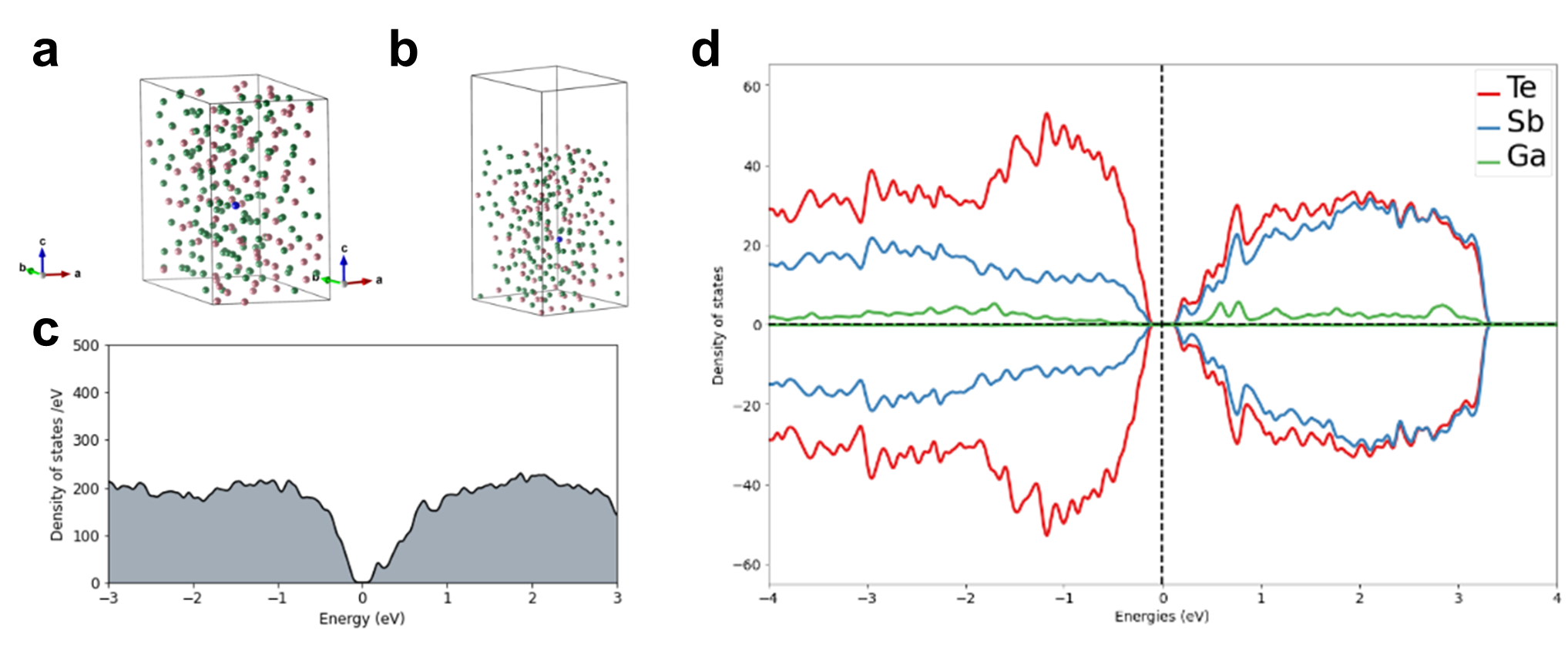


Supplementary Figure 26. (a) Structure of bulk amorphous Sb_2_Te_3_ with a gallium impurity (blue). (b) A surface model including the Ga impurity. c) The density of states of the bulk model including the Ga impurity. (d) Partial density of states for the Ga, Sb, Te in Sb_2_Te_3_ (disregarding spin-orbit mixing). Note the PDOS of the Ga has been multiplied by 20 for readability.

# Model Hamiltonian Calculations

This section presents additional calculations for a simplified model describing the effect of non-magnetic disorder at the surface of a topological insulator, which is taken as a description of the Ga ion-beam effects at the surface of Sb_2_Te_3_. The Hamiltonian and central results of the model are presented in the main text. Here we present additional calculations of the simplified case of defects spread uniformly in a topological insulator and address the existence of both 2D and 1D states that can appear at the A-C boundaries depending on disorder depth.

In the presence of defects, the electron wavefunction of a topological insulator can be localized near the surface, however the resulting states depend closely on the defect density. To examine the effect of defects, a model was constructed of a free-standing slab of TI with finite size in the $xz$ plane and finite along the $y$-direction. It is assumed that the defects are randomly placed in the entire slab with periodic boundary conditions along the $y$-direction. The disorder from each defect is modelled by a scattering potential given by:

$V_{im}\left( r_{i} \right)=\sum_{i=1,N} v_{i}f\left( r-r_{i} \right)$ Supplementary Equation (3)

where $v_{i}$ is the local potential which is assumed to be much larger than the bulk bandgap ^8^, and $f\left( r \right)$ is a range function. The total Hamiltonian is then given by:

$H=H_{TI}+V_{im}$ Supplementary Equation (4)

where $H_{TI}$ is the effective Hamiltonian of three-dimensional topological insulators described in the main text. For Sb_2_Te_3_, the k.p parameters are taken from Ref.^9^ and summarized in the Supplementary Table 5 :

Supplementary Table 5. Sb_2_Te_3_ K.p parameters values used in the calculations.

|  | $m_{0}$ (eV) | $m_{1}$(eV Å^2^) | $m_{2}$ (eV Å^2^) | $A$ (eV Å) | $B$ (eV Å) |
| --- | --- | --- | --- | --- | --- |
| Sb_2_Te_3_ | -0.182 | 22.136 | 51.320 | 3.694 | 1.174 |

To solve the eigenproblem, a multi-band envelope function is used, together with the finite-element method. In Supplementary Figure 27, the electron density for the midgap surface state is depicted for the various values of the defect density.

## Effect of uniform defect density in a topological insulator slab

At low-density, as shown in Supplementary Figure 27 (a) and (b), the wavefunction is still well-localized near the surface, preserving the surface states. In Supplementary Figure 27 (c) and (d), the surface states vanishes with increasing defect density, where electron “bubbles” are formed in the bulk. The TI slab turns into a diffusive metal at moderate defect density (Supplementary Figure 27 (c)), and eventually becomes a normal insulator at high defect density (Supplementary Figure 27 (d)). At the same time, the energy gap ($\Delta_{g})$increases with larger defect-density (Supplementary Figure 27 (e)). The horizontal line indicates the bulk gap of Sb_2_Te_3_ ($\sim0.21$ eV). When the Dirac gap surpasses the bulk gap, the midgap state completely disappears and the system turns into a band insulator/semiconductor. This is in line with the calculated LDOS discussed above.

Furthermore, the conductance of the TI slab can be evaluated by^10^

$G_{TI}=G_{ss}+G_{bulk}$ Supplementary Equation (5)

with

$G_{bulk}=\left[ Ce^{\frac{\Delta_{g}}{k_{B}T}}+D\left( T \right) \right]^{-1}$ Supplementary Equation (6)

$G_{ss}=\left( A+BT \right)^{-1}$ Supplementary Equation (7)

At a finite temperature, the total conductance exponentially reduces with increasing defect density (Supplementary Figure 27 (f)).


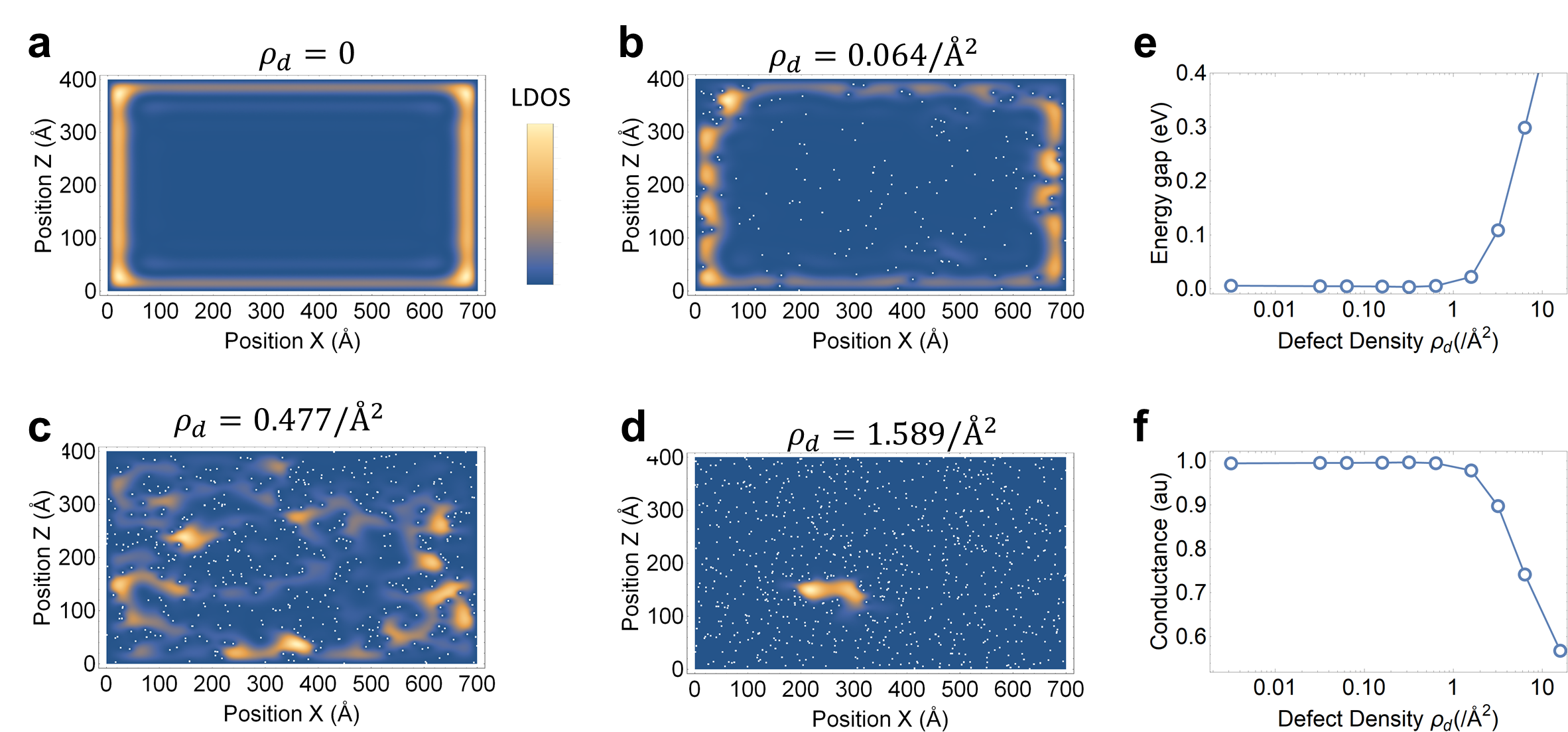
*Supplementary Figure 27. (a-d) Electron density function in a strong topological insulator slab with a uniform distribution of defects throughout the bulk, with different defect densities (ρ_d_). Energy gap (e) and conductance (f) as functions of defect density.*

## Surface state distribution vs. depth of the disorder region.

As the depth of the disorder increases, the surface state (SS) is deformed and ultimately forms an additional 1D edge state at the boundary between the pristine and disordered regions (see Supplementary Figure 28 e). By taking a vertical slice through the slab we can see the surface state initially deflects around the disordered region with an unnoticeable 1D state (Supplementary Figure 28 a). This is ascribed to the fact that the surface state has a finite penetration length (~5nm), so that just a fraction of the surface state is affected when interfacing with an amorphous region with depth smaller than that length.   As the depth of the disorder is increased (Supplementary Figure 28 b and c) a localized state forms near the edge (forming a 1D state) once the depth is comparable to the characteristic penetration length of the SS (~ 5 nm). Eventually the 1D state is predominant over the 2D interface states (c and d) on the top and side surfaces. Further increasing the depth, there are both 1D states and 2D states on the side surface as illustrated schematically in Supplementary Figure 28 e.


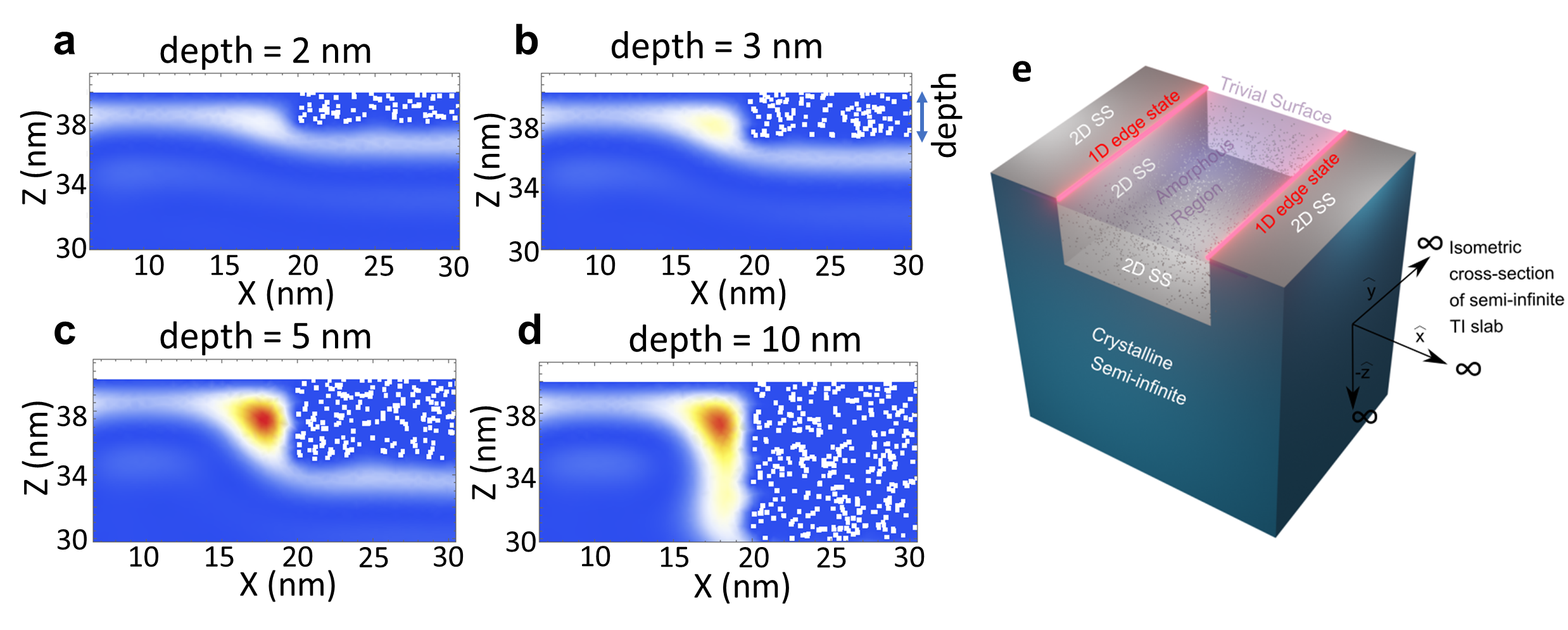


Supplementary Figure 28. Deformation of surface state by disorder. (a) A cross-section of the simulation, showing the surface state (SS) is almost homogeneous around a shallow disordered region. (b) SS is slightly deformed when the disorder region depth increases. (c) A localized state near the edge (1D state) emerges when the depth is comparable to the characteristic penetration length of the SS (~ 5 nm). (d) Further increasing the depth, there are both 1D states and 2D states on the side surface. (e) Illustration of the disordered region embedded at the surface of a semi-infinite topological insulator slab, which hosts both edge states and 2D surface states at the C-A boundary. The co-ordinate system is also shown, as used in a ,b, c, d and the later figures.

## Emergence of 1D edge states

We assume that the disordered region is in the insulating regime so that an open boundary condition can be applied at the boundary. In this case, the Hamiltonian of the system is given by

$H=H_{TI}+V$ Supplementary Equation (8)

with $V=0$ in the crystalline region and $V=\infty$ in the disordered region (modelling a “topological vacuum”).

To simplify the calculation, we also consider periodic boundary conditions along the $y-$direction. The general form of the wavefunction is given by

$\psi\left( x,y,z \right)=e^{ik_{y}y}e^{-\lambda_{x}x}e^{-\lambda_{z}z}$ Supplementary Equation (9)

in which the decay constants in the $x$ and $z$ directions are $\lambda_{x}$ and $\lambda_{z}$, respectively.


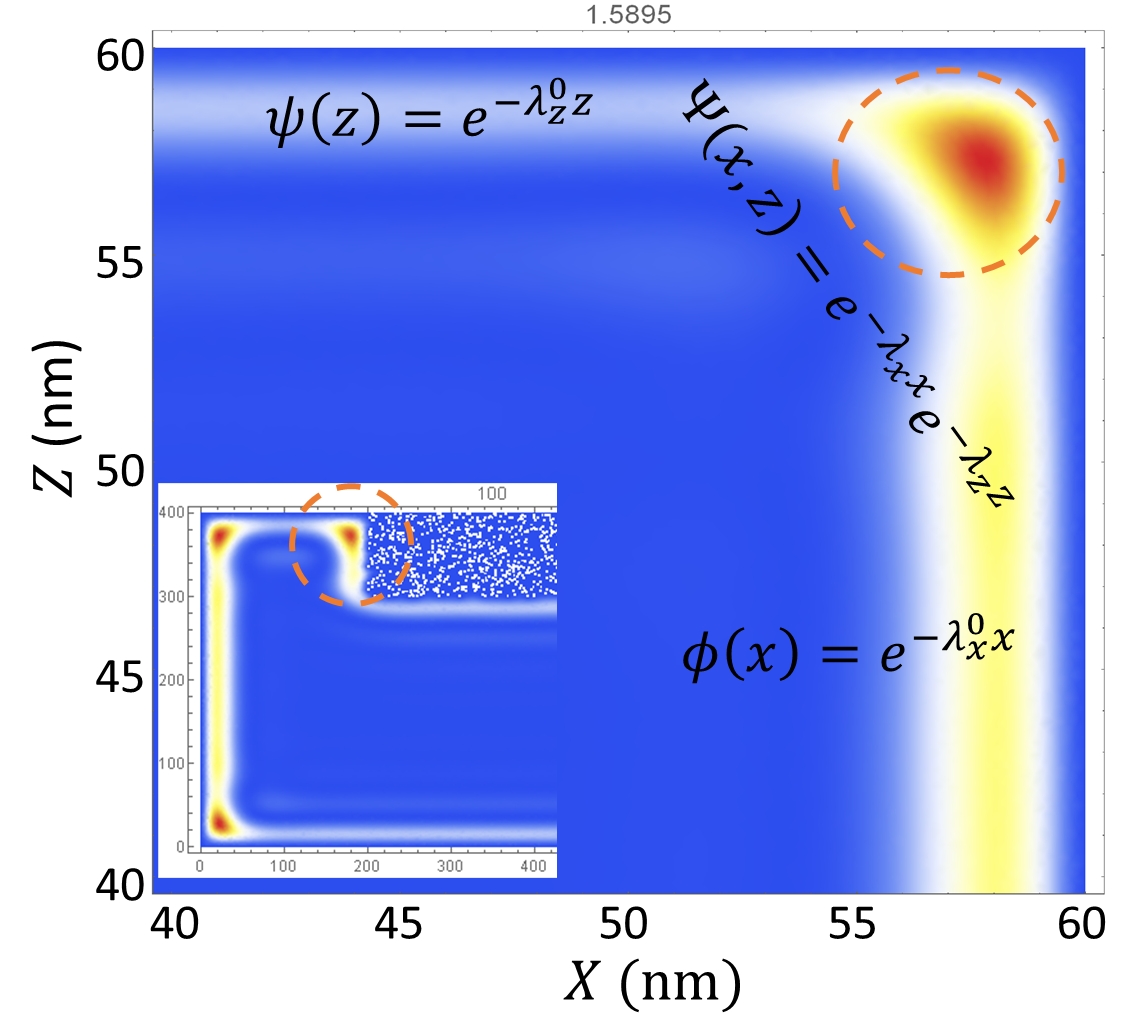


Supplementary Figure 29. The electron density near the boundary corner shows a quasi 1D edge state. The illustration shows the different decay constants for the 1D edge and 2D surface states.

Away from the edge/corner, the decay constants are independent of each other, and they are given by

$\lambda_{z}^{0}=\left[ \frac{\left( B\pm\sqrt{B^{2}+4m_{1}m_{0}} \right)}{2m_{1}} \right], \lambda_{x}^{0}=\left[ \frac{\left( A\pm\sqrt{A^{2}+4m_{0}m_{2}} \right)}{2m_{2}} \right]$ Supplementary Equation (10)

Near the edge, the decay constants are mutually dependent through the following equation:

$A^{2}\lambda_{x}^{2}+B^{2}\lambda_{z}^{2}-\left( m_{1}\lambda_{z}^{2}+m_{2}\lambda_{x}^{2}-m_{0} \right)^{2}=0$ Supplementary Equation (11)

To the first order in $\lambda_{x,z}^{0}$, the decay constants near the edge are obtained as

$$\lambda_{x}^{\pm}=\frac{1}{\sqrt{2m_{2}^{2}}}\left[ A^{2}+2m_{0}m_{2}-2m_{1}m_{2}\left( \lambda_{z}^{0} \right)^{2}\pm\sqrt{A^{2}\left( A^{2}+4m_{0}m_{2} \right)+4\left( \lambda_{z}^{0} \right)^{2}\left( B^{2}m_{2}^{2}-A^{2}m_{1}m_{2} \right)} \right]^{\frac{1}{2}},$$

$$\lambda_{z}^{\pm}=\frac{1}{\sqrt{2m_{1}^{2}}}\left[ B^{2}+2m_{0}m_{1}-2m_{1}m_{2}\left( \lambda_{x}^{0} \right)^{2}\pm\sqrt{B^{2}\left( B^{2}+4m_{0}m_{1} \right)+4\left( \lambda_{x}^{0} \right)^{2}\left( A^{2}m_{1}^{2}-B^{2}m_{1}m_{2} \right)} \right]^{\frac{1}{2}}$$

Supplementary Equation (12)

where $\lambda_{x,z}^{0}$ are given above.

From the above, the top surface exhibits a topological state featuring a 1D edge state near the order/disorder boundary, Supplementary Figure 29. From the decay constants, we can obtain the effective size of the quasi 1D state $l_{x}\times l_{z}=\left( \lambda_{x}\lambda_{y} \right)^{-1}$. With typical values of the parameters for Sb_2_Te_3_, we estimate $l_{x}\approx5 nm$, and $l_{z}\approx5 nm$.

The effective 1D Hamiltonian can be described as follows:

$H_{1D}=Ak_{y}\sigma_{\phi}+\left[ \begin{matrix} 0 & \Omega\\ \Omega^{*} & 0 \end{matrix} \right]$ Supplementary Equation (13)

where $\Omega$ is the spin-Berry phase term acquired by the electron when turning at the corner^11^ . We note here that the spin of the electron does not follow the same spin-momentum locking as on the surfaces$x-$direction as on the top surface or $z-$direction as on the side surface. The electron spin at the corner rather points in the direction determined by an angle $\phi=\arctan\frac{A}{B}$ ^11^, where we recall again that $A$ and $B$ are the Fermi velocities on the top and side surfaces, respectively.

## Effect of geometric imperfections on the 1D/2D boundary states

To study the robustness of the 2D and 1D states, simulations were performed for disorder profiles with soft edges as shown in Supplementary Figure 30. This shows that, while 2D states are robust and wrap the disordered region in all cases, the 1D edge states are more delicate, and can disappear if geometric imperfections are present. Furthermore, the stochastic randomness of the disorder introduces variation in the electron density that leads to regions of both higher and lower density on any edge.


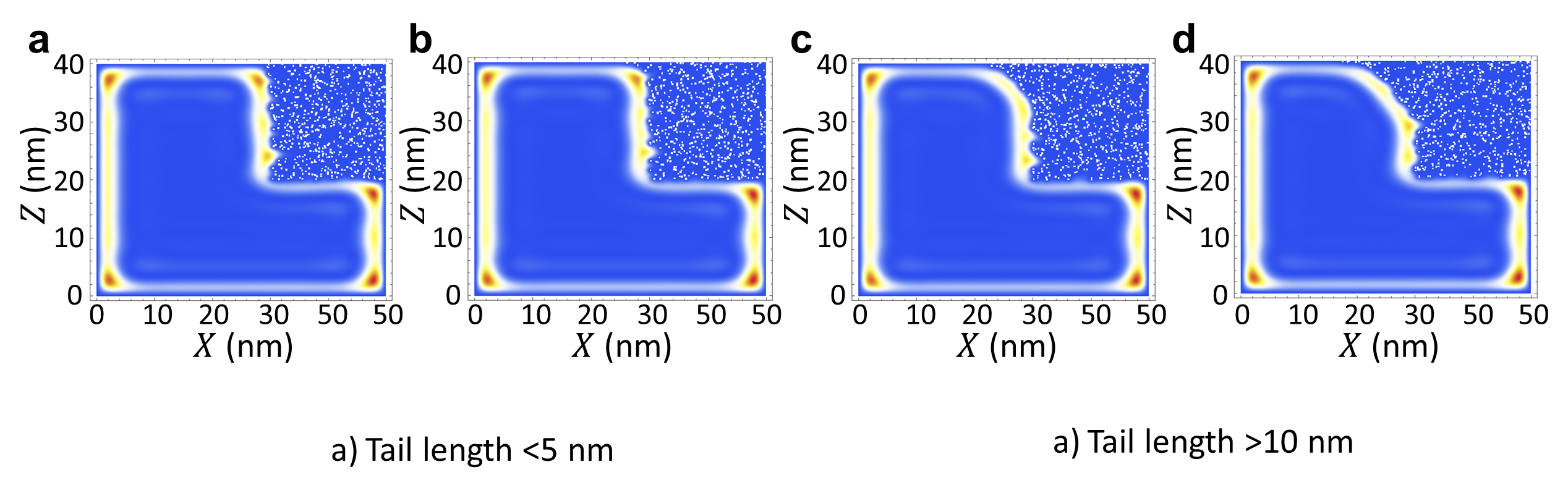


Supplementary Figure 30. Illustration of geometric imperfections in the disordered region with variable tail lengths. This can cause edge states to appear or disappear, depending on the sharpness of the corner. (a) A 1D state is observed at the boundary with a sharp corner. (b-d) The 1D state progressively disappears as the corners become more rounded.

## Predicted effect of magnetic field on the topological states

Magnetic fields are a useful tool to differentiate between the 1D helical and 2D states at the A-C boundary, both of which potentially appear at the A-C boundary. We have performed preliminary theory, showing how the two different scenarios respond to fields, by using the following Hamiltonian:

$H=H_{\mathrm{TI}}+H_{Z}\otimes\mathbb{I}_{2}$ Supplementary Equation (14)

$H_{Z}=g_{\parallel}{\mu_{B}\sigma}_{z}B_{z}+g_{\perp}\mu_{B}{(\sigma}_{x}B_{x}+\sigma_{y}B_{y})$ Supplementary Equation (15)

$H_{\mathrm{TI}}$ is the Hamiltonian described earlier for the disordered topological insulator, whereas $H_{Z}$is the magnetic field term, and $\mathbb{I}_{2}$ is the identity matrix. The g-factors in Sb_2_Te_3_ are estimated to be in the range of g~20$-$30 ^12^.

A Zeeman splitting is induced when a magnetic field is applied perpendicular to the surface of the TI. For $B_{z}=7 T$, the Zeeman splitting is about $\Delta E_{Z}=8-12$ meV.The results depend on the field direction and magnitude as shown in the Supplementary Figure 31. Generally, the 2D states survive; however, the 1D states can be removed by applying the field in specific directions. However, it has not yet been possible to verify this experimentally as it would require a substantial redesign of the devices and apparatus, but this will be the target of future work.


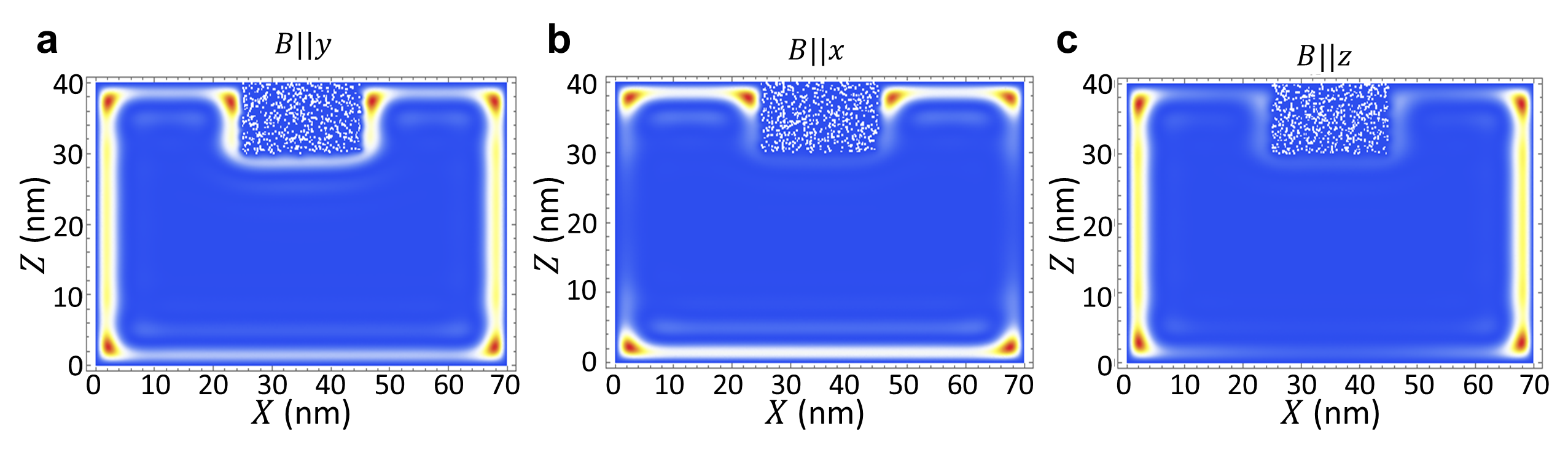


Supplementary Figure 31. Surface states (SS) distribution in the presence of a magnetic field. (a) A magnetic field applied along the y-direction. (b) A magnetic field applied along the x-direction will destroy the SS on the planes perpendicular to the x-direction. However, we can still observe the 1D states around the disorder region. (c) A perpendicular magnetic field B_z_ (along z-direction) will destroy both 2D surface states and 1D corner states. In the above calculations, magnetic field B=7 T, and an isotropic g-factor = 30 is assumed.

**Supplementary References**

1 Ziegler, J. F., Ziegler, M. D. & Biersack, J. SRIM–The stopping and range of ions in matter. *Nuclear Instruments and Methods in Physics Research Section B: Beam Interactions with Materials and Atoms* **268**, 1818-1823 (2010).

2 Hengstler-Eger, R. *et al.* Heavy ion irradiation induced dislocation loops in AREVA’s M5® alloy. *J. Nuclear Mater.* **423**, 170-182 (2012).

3 Frey, L., Lehrer, C. & Ryssel, H. Nanoscale effects in focused ion beam processing. *J. Appl. Phys. A* **76**, 1017-1023 (2003).

4 Bake, A. *et al.* Lamellae preparation for atomic-resolution STEM imaging from ion-beam-sensitive topological insulator crystals. *Journal of Vacuum Science & Technology A: Vacuum, Surfaces, and Films* **40**, 033203 (2022).

5 Zhao, W. *et al.* Quantum oscillations in iron-doped single crystals of the topological insulator Sb_2_Te_3_. *Physical Review B* **99**, 165133 (2019).

6 Rauh, H., Geick, R., Kohler, H., Nucker, N. & Lehner, N. Generalized phonon density of states of the layer compounds Bi_2_Se_3_, Bi_2_Te_3_, Sb_2_Te_3_ and Bi_2_(Te_0.5_Se_0.5_)_3_, (Bi_0.5_Sb_0.5_)_2_Te_3_. *Journal of Physics C: Solid State Physics* **14**, 2705 (1981).

7 Grimme, S., Ehrlich, S. & Goerigk, L. Effect of the damping function in dispersion corrected density functional theory. *Journal of Computational Chemistry* **32**, 1456-1465 (2011).

8 Schubert, G., Fehske, H., Fritz, L. & Vojta, M. Fate of topological-insulator surface states under strong disorder. *Phys. Rev. B* **85**, 201105 (2012).

9 Nechaev, I. A. & Krasovskii, E. E. Relativistic *k.p* Hamiltonians for centrosymmetric topological insulators from ab initio wave functions. *Phys. Rev. B* **94**, 201410 (2016).

10 Corbae, P. *et al.* Evidence for topological surface states in amorphous Bi_2_Se_3_. *arXiv preprint arXiv:.13412* (2019).

11 Imura, K.-I., Takane, Y. & Tanaka, A. Spin Berry phase in anisotropic topological insulators. *Physical Review B* **84**, 195406 (2011).

12 Korzhovska, I. *et al.* Spin memory of the topological material under strong disorder. *npj Quantum Materials* **5**, 39 (2020).
